# Investigating Native Metal Ion Binding Sites in Mammalian Histidine-Rich Glycoprotein

**DOI:** 10.1021/jacs.3c00587

**Published:** 2023-03-31

**Authors:** Katrin Ackermann, Siavash Khazaipoul, Joshua L. Wort, Amélie I. S. Sobczak, Hassane El Mkami, Alan J. Stewart, Bela E. Bode

**Affiliations:** † EaStCHEM School of Chemistry, University of St Andrews, North Haugh, St Andrews KY16 9ST, Scotland; ‡ Biomedical Sciences Research Complex, University of St Andrews, North Haugh, St Andrews KY16 9ST, Scotland; § Centre of Magnetic Resonance, University of St Andrews, North Haugh, St Andrews KY16 9ST, Scotland; ∥ School of Medicine, University of St Andrews, North Haugh, St Andrews KY16 9TF, Scotland; ⊥ School of Physics and Astronomy, University of St Andrews, North Haugh, St Andrews KY16 9SS, Scotland

## Abstract

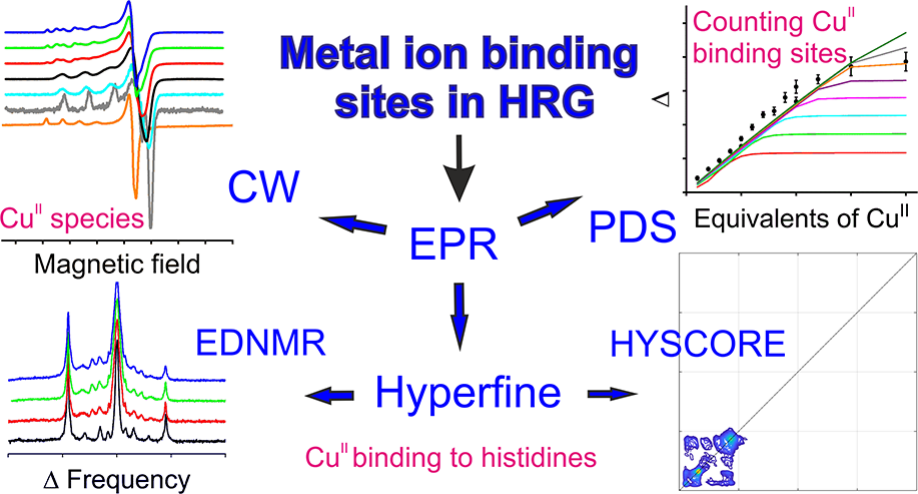

Mammalian histidine-rich glycoprotein (HRG) is a highly versatile and abundant blood plasma glycoprotein with a diverse range of ligands that is involved in regulating many essential biological processes, including coagulation, cell adhesion, and angiogenesis. Despite its biomedical importance, structural information on the multi-domain protein is sparse, not least due to intrinsically disordered regions that elude high-resolution structural characterization. Binding of divalent metal ions, particularly Zn^II^, to multiple sites within the HRG protein is of critical functional importance and exerts a regulatory role. However, characterization of the Zn^II^ binding sites of HRG is a challenge; their number and composition as well as their affinities and stoichiometries of binding are currently not fully understood. In this study, we explored modern electron paramagnetic resonance (EPR) spectroscopy methods supported by protein secondary and tertiary structure prediction to assemble a holistic picture of native HRG and its interaction with metal ions. To the best of our knowledge, this is the first time that this suite of EPR techniques has been applied to count and characterize endogenous metal ion binding sites in a native mammalian protein of unknown structure.

## Introduction

In this report, we derive a holistic picture of intrinsic metal ion binding sites in a native mammalian plasma protein obtained from electron paramagnetic resonance (EPR) spectroscopy supported by computational protein structure prediction. No high-resolution experimental structure of the full-length protein has been reported to date.

Mammalian histidine-rich glycoprotein (HRG) is a glycosylated protein of ∼70 kDa in size and is present in blood plasma at relatively high concentrations (∼1.5 μM).^1^ It has numerous binding partners, such as heparin, plasminogen, divalent metal ions, and heme, and is involved in many essential regulatory biological processes, including blood coagulation, cell migration, proliferation and adhesion, tumor growth inhibition and angiogenesis, as well as immune complex clearance.^1−7^ HRG has therefore been referred to as the “Swiss Army knife of mammalian plasma”.^8^

**Figure 1:**
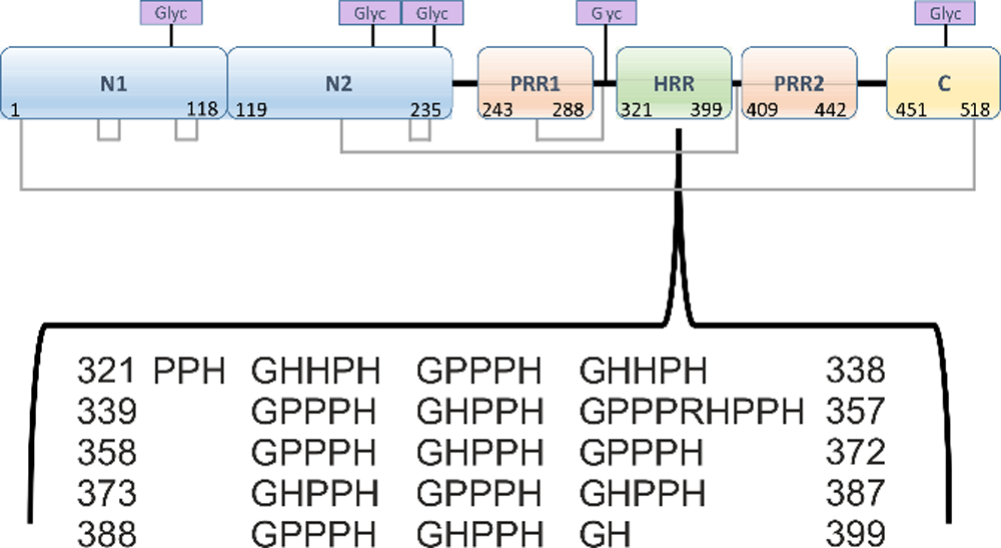
Domain structure of rabbit HRG showing the disulfide bridging arrangements (gray lines, disulfide bonds at positions 6–497, 60–71, 87–108, 185–407, 199–222, and 264–294) and five putative glycosylation sites (predicted asparagine glycosylation sites at positions 107, 184, 232, 302, and 477, indicated by purple boxes). Residue numbers are given with respect to the mature protein, i.e., without the N-terminal signal sequence (comprising the N-terminal eight residues before cleavage). The inset details the primary structure of the HRR (amino acid sequence for residues 321–399), with tandem repeats of the consensus sequence G[H/P][H/P]PH.

Despite a plethora of functions, there is surprisingly little experimental data relating to the structure of HRG. The protein exhibits a multi-domain arrangement with two N-terminal (N1 and N2) and one C-terminal (C) domain and has a central histidine-rich region (HRR) that is flanked by proline-rich regions (PRRs) on either side (Figure 1). While this domain structure is well established, a high-resolution structure is only available for one of the two N-terminal domains (N2).^9^ The small size of HRG makes cryo-electron microscopy rather challenging, and an HRG fragment comprising the HRR and parts of both PRRs was predicted to be intrinsically disordered,^5^ which may explain the lack of available high-resolution X-ray structures for the full-length protein.^9,10^ In fact, recent availability of AlphaFold2 (AF2) protein structure prediction^11,12^ in combination with JPred4 secondary structure prediction^13^ based on the full HRG sequence strongly supports the idea of high disorder (in the absence of interaction partners) for the PRR1-HRR-PRR2 stretch (Figures S1 and S2).

Several functions of HRG have been described to be mediated by Zn^II^.^3,6,7^ In the physiological context, free Zn^II^ is cytotoxic at low concentrations; thus, plasma Zn^II^ levels are tightly regulated, with the majority of the 15–20 μM Zn^II^ present in plasma bound to (mostly) albumin and small molecules, keeping the free Zn^II^ concentration in the nanomolar range.^1,14^ Within the organism, increased Zn^II^ levels can arise following release from activated platelets or damaged cells,^15^ reaching transient localized Zn^II^ concentrations estimated to be up to 50 μM, allowing binding to effector proteins such as HRG.^1,14^ Zn^II^ binding to HRG was demonstrated to exert a regulatory effect on HRG binding to other targets and ligands by modifying respective affinities.^1,9,15^ The large number of histidine residues makes the HRR the primary suspect for binding the divalent metal ions.^1^ Early binding studies reported sites accommodating approximately 10 to 20 divalent metal ions,^16,17^ and a more recent study confirmed the binding of 10 Zn^II^ ions using isothermal titration calorimetry (ITC).^3^ This number is consistent with approximately 12 tandem repeats of the consensus sequence G[H/P][H/P]PH present within the HRR in HRG (13 repeats in rabbit, see Figure 1),^18−20^ which can function as high-affinity metal ion binding sites by offering coordination by multiple histidine residues.^1,21,22^ However, a detailed understanding of the local environment of the metal ion binding sites is lacking.

Currently, mammalian plasma is the main source of pure HRG protein. This has allowed only limited systematic investigation of metal ion binding.^3,5,17,23^ Furthermore, since we presently cannot produce natively glycosylated HRG recombinantly to sufficient yield, routine modifications performed with in vitro protein production such as site-directed mutagenesis and selective or uniform labeling with e.g., isotopes or spin labels (see below) are out of reach for the time being.^10^

EPR is an important tool for structural analysis and characterization of biomacromolecules. In contrast to other structural biology methods, EPR is not limited by the size, shape, or complexity of the system and does not require protein crystallization. Compared to techniques such as ITC, less material is required, and samples can be stored frozen and reused. EPR measurements require the presence of paramagnetic species, which can be either endogenous, such as paramagnetic metal centers or cofactors, or deliberately introduced to the site(s) of interest. The latter is commonly achieved by incorporating stable nitroxide radicals via site-specific mutagenesis and site-directed spin labeling^24^ or by site-specifically engineering artificial metal ion binding sites.^22,25^ Another option is substituting endogenous diamagnetic metal ions (e.g., Zn^II^) with paramagnetic ones (e.g., Cu^II^).^26,27^

Different EPR techniques provide access to local structural information around paramagnetic centers,^28−34^ nanometer distances between those centers employing pulse dipolar EPR spectroscopy (PDS),^35−47^ and binding constants.^48−50^ Continuous wave (CW) EPR can be used to characterize the binding geometry around the paramagnetic metal center and to resolve interactions with nearby nuclear spins, including superhyperfine (SHF) interactions with directly coordinating nitrogen atoms.^51,52^ Hyperfine spectroscopy can identify the orbitals harboring the unpaired electron and their distance to close-by magnetic nuclei; this is useful to investigate whether different binding sites are sequentially populated (in contrast to all sites having similar binding constants). One hyperfine method is ELDOR-detected NMR (EDNMR),^53,54^ which provides information on magnetic nuclei directly coordinated to (and thus strongly coupled to) the paramagnetic center.^53,54^ Complementarily, electron spin echo envelope modulation (ESEEM) is sensitive to weakly coupled nuclei and can therefore help elucidate interactions between paramagnetic centers and more distant nuclear spins.^33,55,56^ These experiments allow, for example, assessment of the number of remote amino nitrogen atoms attributed to histidine residues present at metal ion binding sites.^57,58^ The corresponding hyperfine sublevel correlation (HYSCORE)^56,59^ experiment essentially adds a second dimension to the ESEEM experiment, and the resulting cross-peaks can attribute electron-nuclear couplings to electron spin centers and ease data interpretation.^28,34,60^ To complement these hyperfine techniques, PDS experiments, such as the pulsed electron–electron double resonance method (PELDOR aka DEER), are commonly used to determine distances between paramagnetic centers on the nanometer scale.^43,61−63^ Furthermore, the number of spins or paramagnetic centers present per complex is encoded in the PELDOR modulation depth (Δ),^64,65^ which is also the basis for determination of binding constants.^48−50^

In the present work, we employed EPR toward the characterization of metal ion binding sites and their local environment in HRG purified from rabbit plasma to enhance our understanding of the role of metal ions in HRG-regulated biological processes. We first established that Cu^II^ can be used as a proxy for Zn^II^ to enable CW and pulse EPR studies employing ITC and binding assays. Importantly, a recent report found that HRR-derived peptides not only bind Cu^II^ but may influence copper homeostasis,^66^ indicating the potential physiological relevance of Cu^II^ binding to HRG. Using a suite of EPR techniques including CW and hyperfine spectroscopy, we then characterized the local environment and topology of Cu^II^ ions bound to native HRG. PDS measurements were performed to investigate the global arrangement and potential preferential order of the Cu^II^ binding sites within the HRG protein. Here, a speciation model for the occupation of high- and low-affinity metal ion binding sites was developed based on PELDOR modulation depths. Together with structure predictions, a holistic picture of HRG binding to divalent metal ions emerged with a potential regulatory role in HRG function.

## Results and Discussion

**Figure 2:**
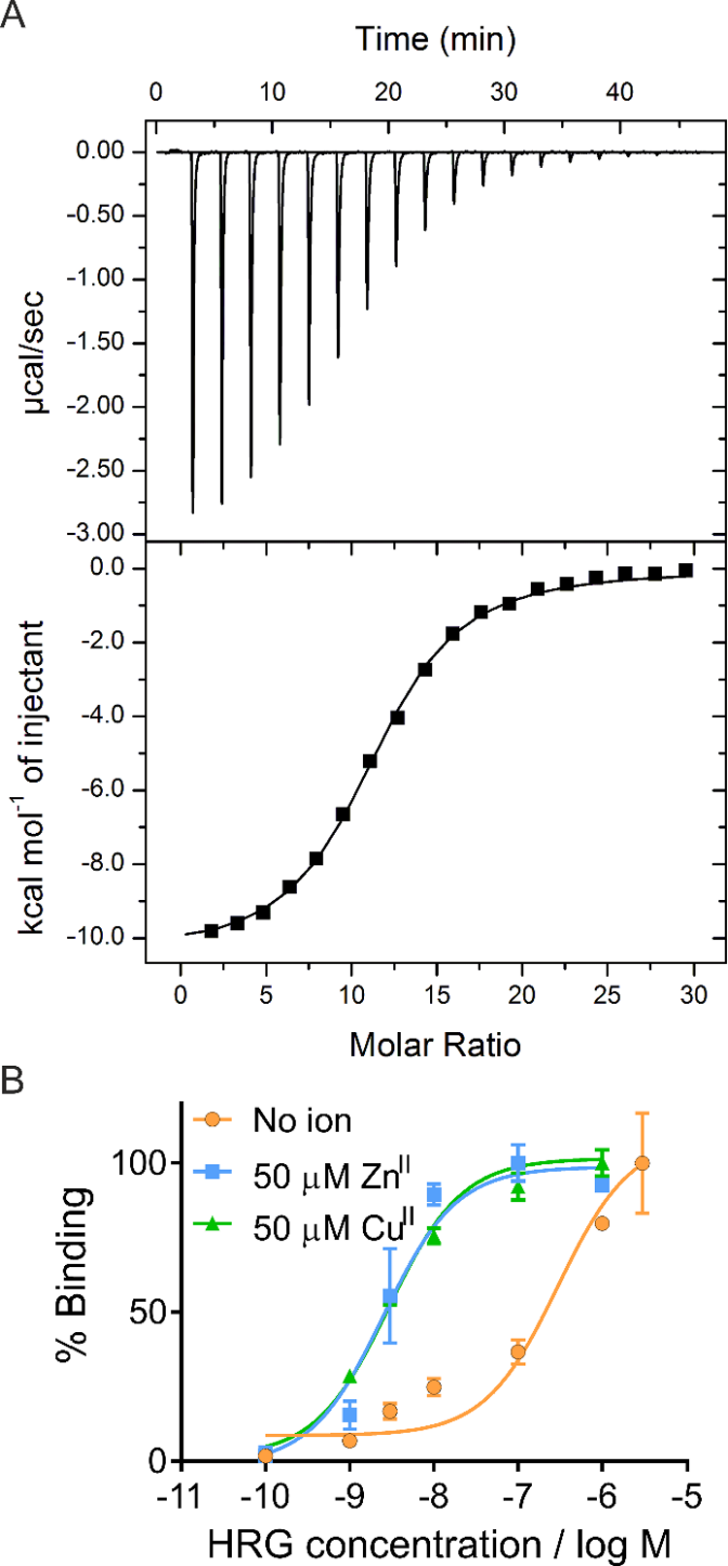
(A) ITC data for Cu^II^ binding to HRG. The upper panel shows the resultant raw data, and the lower panel shows the fitted data. Raw data were fitted using a “one-set-of-sites” model in Origin 7.0. From the resultant fit, the number of binding sites *N* and the dissociation constant (*K*_ITC_) were determined as *N* = 11.1 ± 0.1 sites and *K*_ITC_ = (6.3 ± 0.4) × 10^–6^, respectively. (B) Effect of Zn^II^ and Cu^II^ on binding of rabbit HRG to immobilized unfractionated heparin. Assays were performed in triplicate; error bars represent the SEM. The resultant dissociation constants (*K*_D_) obtained were (2.98 ± 0.75) × 10^–7^ in the absence of metal ions, (2.5 ± 0.5) × 10^–9^ in the presence of 50 μM Zn^II^, and (2.9 ± 0.5) × 10^–9^ in the presence of 50 μM Cu^II^.

EPR spectroscopy requires the presence of paramagnetic species in the sample. Binding of diamagnetic Zn^II^ can therefore not be examined by EPR directly; however, previous studies of HRG or HRG-derived peptides have reportedly used the paramagnetic Cu^II^ instead.^17,23^ To confirm the validity of this approach, we demonstrated that ITC yields very similar numbers of binding sites and binding affinities in vitro when comparing paramagnetic Cu^II^ (this study, Figure 2A) and diamagnetic (EPR-silent) Zn^II^.^3^ Next, a heparin binding assay confirmed a similar increase in the affinity of HRG binding to heparin for both, Zn^II^ and Cu^II^, indicating the exertion of a similar functional effect (Figure 2B). Together, ITC and heparin binding data suggested that Cu^II^ is a promising proxy for Zn^II^ for performing EPR spectroscopic studies of the local environment of metal ion binding sites and their spatial distribution in HRG. For details on sample preparation and experimental procedures, see Supplementary Information (SI).

**Figure 3:**
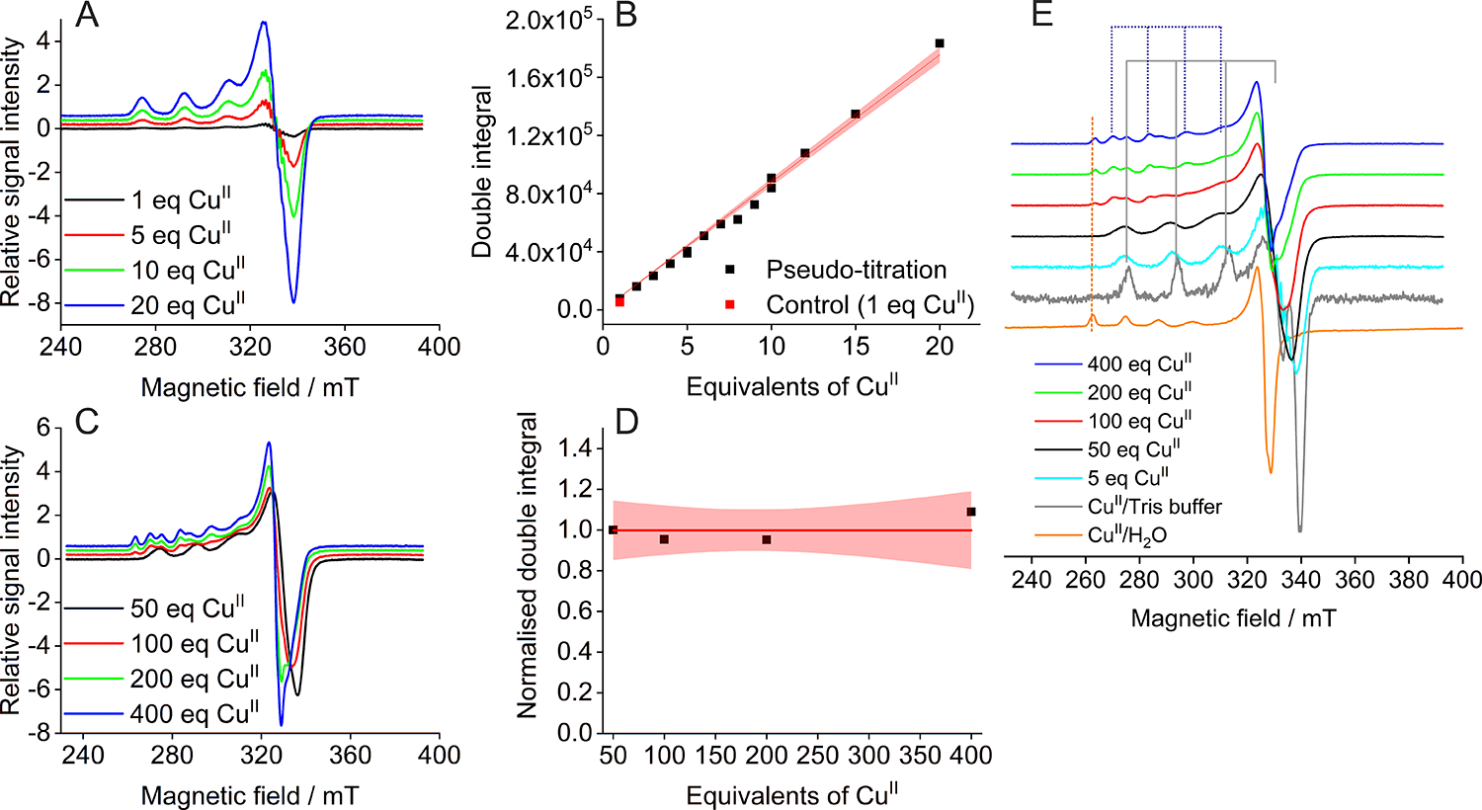
CW EPR spectra. Top row: “Pseudo-titration” series. Two batches of protein were used, and samples at 5 and 10 molar equivalents of Cu^II^ were prepared from both batches to test reproducibility. (A) Stacked overlays of selected individual CW EPR spectra from the pseudo-titration series. (B) Quantification by double integrals obtained from the pseudo-titration samples. Linear fit with the 95% confidence band is shown in red. Bottom row: High-load Cu^II^ samples. (C) Stacked individual CW EPR spectra. (D) Corresponding double integrals (all samples have 2.5 mM Cu^II^). Constant fit with the 95% confidence band in red. (E) Stacked overlays of normalized CW EPR spectra for samples at 5 molar equivalents of Cu^II^ (625 μM Cu^II^) and high Cu^II^ loading (all samples have 2.5 mM Cu^II^) and control samples as indicated in the legend for comparison of observed Cu^II^ species. Solid gray and dotted blue lines indicate the A_II_ hyperfine splittings for Cu^II^ species involving nitrogen in the coordination environment. From 100 molar equivalents of Cu^II^, an additional spectral component could be observed in the HRG samples corresponding to the species observed in the Cu^II^/water control.

For EPR investigations of HRG, a “pseudo-titration” series^49,64^ (each titration point is a discrete sample) was prepared, keeping the HRG concentration constant (125 μM final protein concentration) and adding Cu^II^ in the range from 1 to 20 molar equivalents (125 μM to 2.5 mM). Frozen solution CW EPR spectra were recorded for each sample to observe the Cu^II^ species present (Figures 3A, S3–S5). The continuous, almost linear increase in the Cu^II^ signal from 1 to 20 molar equivalents of Cu^II^ (Figure 3B) indicated that the Cu^II^ observed in the spectrum accounted for virtually all Cu^II^ added, excluding potential precipitation or anti-ferromagnetic interaction that would have resulted in a reduced Cu^II^ signal.

Resolved SHF couplings could be assigned to directly ligating nitrogen atoms. These ^14^N SHF splittings remained clearly visible from the initial addition of 1 up to ∼10–12 molar equivalents of Cu^II^ (Figure S6). This suggested the absence of coordinatively distinct metal ion binding sites with substantially different SHF coupling patterns. Thus, CW EPR data nicely complemented the presented ITC results.^3,16^ While ITC showed the presence of 10–12 high-affinity Cu^II^ binding sites in HRG, CW EPR demonstrated that the first 10–12 Cu^II^ ions were bound to nitrogen ligands. In the context of HRG, these sites are tentatively assigned to histidine sidechains in the HRR.

The resolution of ^14^N SHF coupling gradually decreased upon addition of Cu^II^, in agreement with earlier data.^17^ This can either be caused by spectral broadening by the interaction between Cu^II^ ions in close proximity or by a variety of sites being occupied (but not merely “diluting out” the SHF signal, see SI for more detailed discussion). To further investigate the appearance of additional, lower-affinity metal ion binding sites and determine the maximum binding capacity of HRG, a set of samples with high Cu^II^ loading (from 50 to 400 molar equivalents) was assessed (Figure 3C). Here, the protein concentration was varied (from 6.25 to 50 μM), while the Cu^II^ concentration was kept constant (at 2.5 mM), yielding a constant double integral of the resulting CW signal, within the confidence estimate (Figure 3D).

Importantly, while the 50-equivalent Cu^II^ sample showed mainly broadening in the CW spectrum compared to the 5-equivalent Cu^II^ sample, the higher Cu^II^ equivalent samples clearly demonstrated the appearance of additional Cu^II^ species by stark changes in the spectral lineshapes (Figure 3E). Comparing g_II_ and A_II_ values retrieved from the CW spectra (Table S1) to Peisach–Blumberg correlations^29^ and recent reports on nitrogen (histidine/imidazole)-coordinated Cu^II^,^28,32^ our data suggested multiple Cu^II^ coordination modes. Initially, Cu^II^ was bound by as many as four nitrogen ligands (histidine residues; Tris buffer, see the SI chapter “EPR sample preparation” for discussion), while higher excess of Cu^II^ led to coordination involving both nitrogen and oxygen atoms (2N2O; 3N1O) as well as the appearance of free Cu^II^ (see spectrum of Cu^II^ in water), indicating that the maximum binding capacity of HRG was exceeded. These findings have a direct structural impact on HRG as they demonstrated a Cu^II^ concentration-dependent shift in Cu^II^ coordination, implying that induced structural changes in HRG are required for, and depend on, the metal loading.

**Figure 4:**
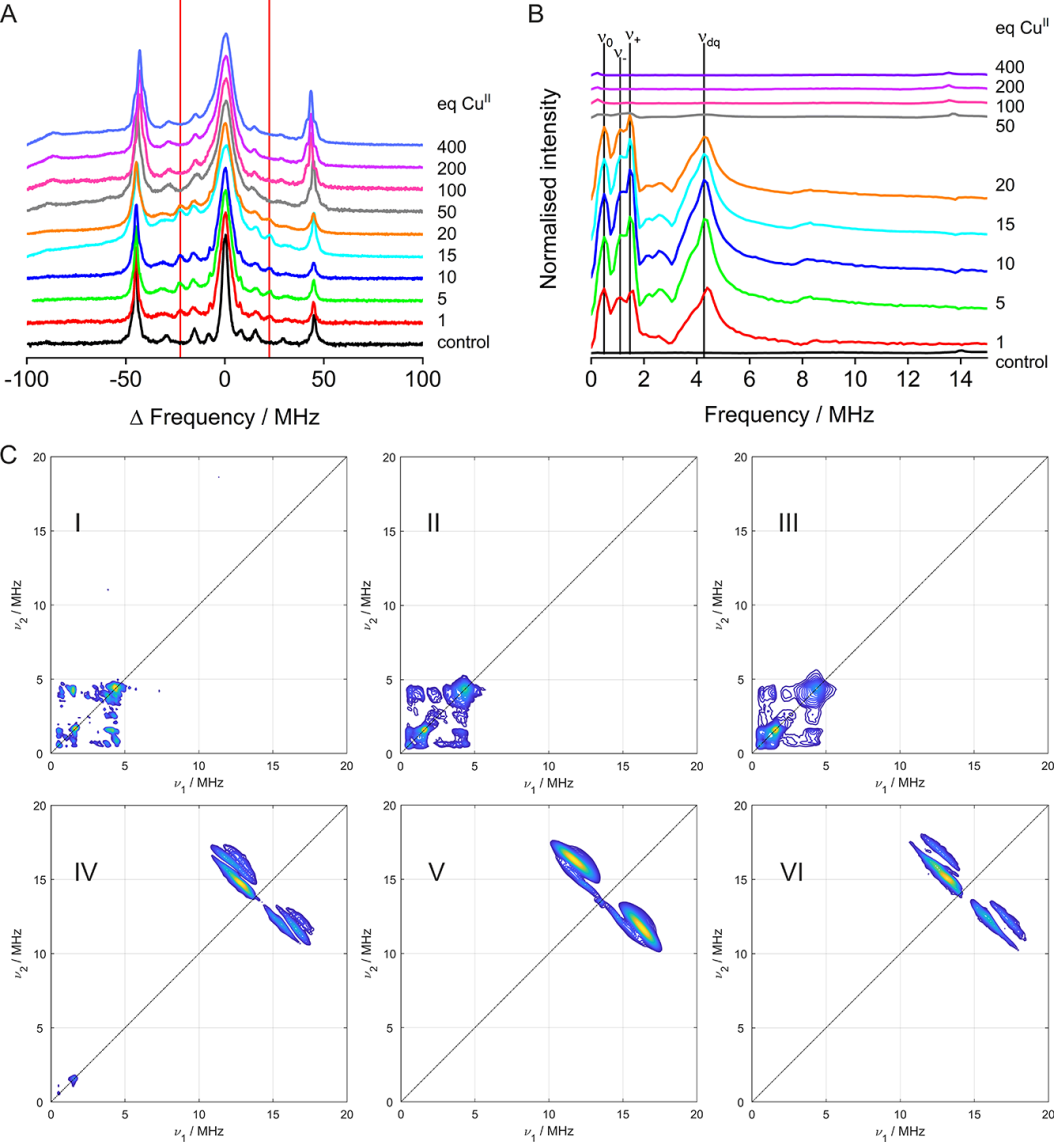
Hyperfine spectroscopy. (A) Stacked plot of EDNMR spectra obtained at Q-band frequencies (34 GHz) and low field (1.0240–1.0630 T) for increased resolution. Vertical lines indicating the additional peaks appearing in the presence of HRG are shown in red. (B) Stacked plots of three-pulse ESEEM data obtained at X-band frequencies (9.5 GHz) and on the maximum of the field-swept spectra. Vertical lines indicate the different frequencies to be expected in the presence of two histidine residues (NQI: ν_0_, ν_+_, ν_–_, DQ: ν_dq_); these were employed for quantitative analysis. (C) (+,+) HYSCORE spectra for 1 (I), 5 (II), 20 (III), 50 (IV), and 400 (V) molar equivalents of Cu^II^ and control (VI; 1 equiv Cu^II^ in Tris buffer), obtained at X-band frequencies (9.5 GHz) and on the maximum of the field-swept spectra.

The question of whether the gradual loss of SHF resolution in the CW spectra was due to inhomogeneous line broadening (while having the same underpinning SHF coupling) was investigated using hyperfine spectroscopy. In the presence of HRG, EDNMR spectra displayed additional peaks compared to Cu^II^ in buffer (Figures 4A and S7, Table S2), confirming coordination by at least two imino nitrogen nuclei of imidazole rings to Cu^II^. These were tentatively assigned to histidine residues forming the Cu^II^ binding sites in the HRR. The hyperfine structure was virtually unchanged from 1 up to 15–20 molar equivalents of Cu^II^. Relating these results to the frozen CW data, the disappearance of the ^14^N SHF concomitant with line broadening could not be explained by the appearance of additional couplings, which would have given rise to additional peaks in the EDNMR. Instead, it could be concluded that dipolar line broadening because of electron–electron interactions caused by close proximity of binding sites led to the gradual loss of resolutionof the SHF coupling.

Furthermore, higher Cu^II^ loading (≥50 molar equivalents) led to the complete loss of the additional peaks in the EDNMR spectra, suggesting that other (non-histidine) binding sites have become dominant, which must be of lower affinity due to their occupation only at higher Cu^II^ concentrations. These data indicated that the different binding sites may have different relaxation behavior, leading to the hypothesis that Cu^II^ bound to high-affinity sites relaxes faster and thus vanishes from the signal once lower-affinity sites are occupied (see SI for more detailed discussion).

ESEEM spectra up to 20 molar equivalents of Cu^II^ showed very similar frequencies (within experimental uncertainty) typically observed for remote nitrogen atoms at 9–10 GHz electron Larmor frequency and were assigned to nuclear quadrupole interactions (NQI, several narrow lines in the range of ∼0.5–2 MHz) and the ^14^N double quantum transition (DQ, a broader line around 4 MHz) (Figures 4B and S8).^56,57,67^ These results were in line with the EDNMR data, indicating that the involved histidine binding sites were very similar (or the same sites were filled gradually). Importantly, while favorable cases may allow extraction of information on tensors and bond lengths and angles,^67^ this approach is not feasible for HRG due to its putative structural heterogeneity and the large number of equivalent but non-identical binding sites. Quantitative analysis of ESEEM experiments provided further support for our EDNMR data (Figures S9 and S10, Table S3). Here, changes in the DQ peak integral, which directly depends on the number of histidine residues involved in the binding site,^57^ suggested coordination of the Cu^II^ by at least two imidazole rings for up to 15 molar equivalents of Cu^II^ and less histidine residues per Cu^II^ available for binding at higher Cu^II^ loading (see SI for more detailed discussion).^31,57^ Importantly, ESEEM dropped off significantly at higher Cu^II^ loading (≥50 molar equivalents), which was consistent with EDNMR data.

Results were further corroborated by the corresponding two-dimensional HYSCORE experiments demonstrating the same shape and combination peaks from 1 to 20 molar equivalents of Cu^II^, strongly suggesting that highly similar binding sites were occupied. Consistent with ESEEM results, HYSCORE spectra changed substantially at higher molar equivalents of Cu^II^, resembling the control spectrum (Tris-/water-coordinated Cu^II^), indicating that new non-histidine sites were occupied. These sites displayed strong interaction with water protons and were thus more solvent exposed than the higher-affinity sites in the HRR (Figures 4C, S11–S12).

Taken together, all hyperfine spectroscopy results suggested similar environments for the high-affinity binding sites, involving at least two imidazole rings per site, and the presence of further lower-affinity metal ion binding sites independent of histidine residues. Importantly, occupation of these lower-affinity sites eliminated observed imidazole-linked hyperfine couplings, further supporting the hypothesis of significantly different relaxation behavior for the different sites.

**Figure 5:**
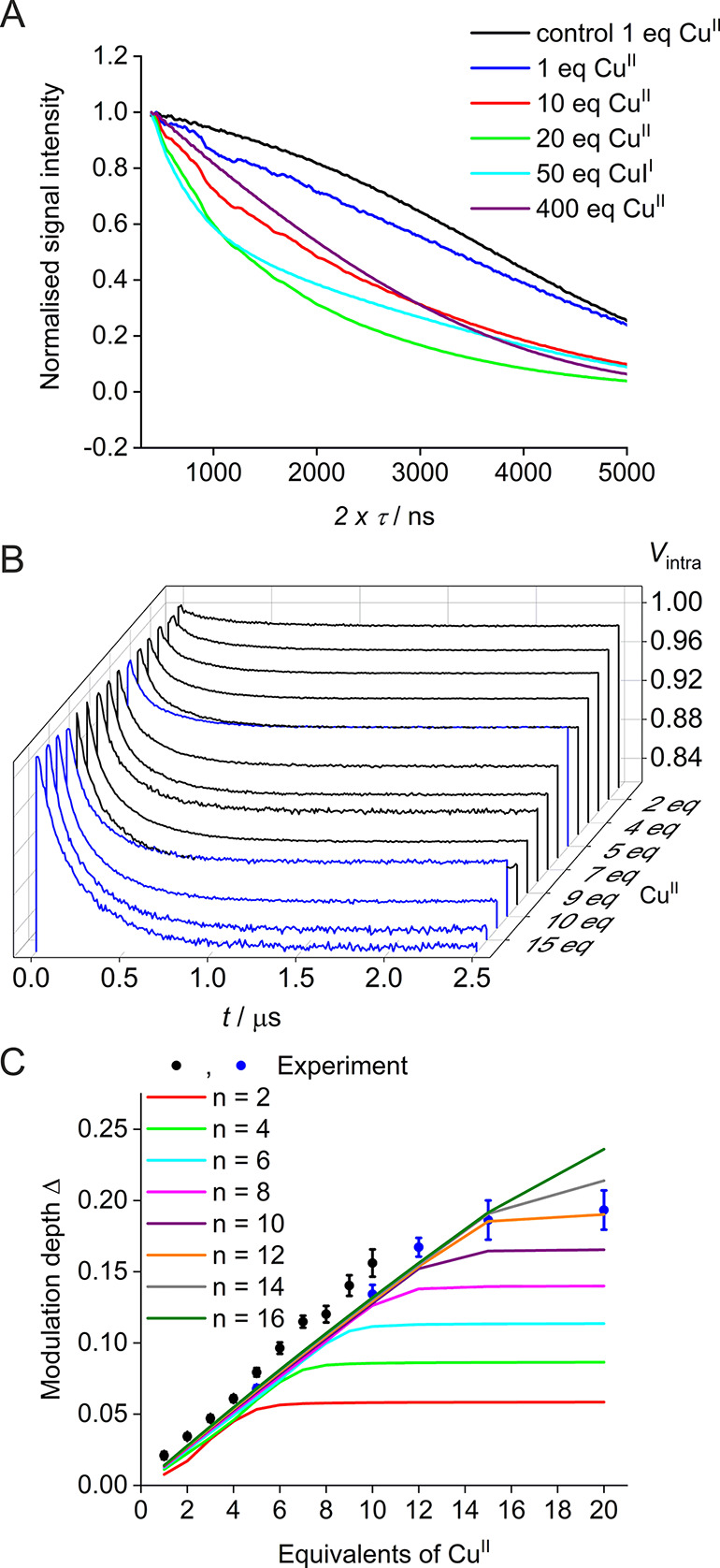
(A) Two-pulse decay for selected samples (zoom-in, for the full *x* axis, see Figure S13). (B) Waterfall plot of background-corrected PELDOR traces (for raw traces, see Figure S15) obtained from the pseudo-titration series of HRG and Cu^II^. Samples were either prepared from the initial batch of purified HRG (black lines) or from the second batch of HRG (blue lines). (C) Corresponding mean modulation depths (±2 × σ error) as obtained from the pseudo-titration series of HRG and Cu^II^. Samples containing 1 to 10 molar equivalents of Cu^II^ (black scatter) were prepared from the initial batch of purified HRG. A second batch of HRG was purified to further increase the equivalents of Cu^II^ (blue scatter) to observe saturation of the high-affinity binding sites. Solid lines show simulations of PELDOR modulation depths based on the speciation model corresponding to values of *n* = [2, 4, 6, 8, 10, 12, 14, 16]. (Simulations were performed with the following other parameters: [*P*]_0_ = 1.25 × 10^–4^ M, [*M*]_0_ = [0.125, 0.250, 0.375, 0.500, 0.675, 0.750, 0.875, 1.000, 1.125, 1.250, 1.500, 1.875, 2.500] × 10^–3^ M, *K*_D1_ = 5 × 10^–8^, *m =* 3, *K*_D2_ = 10 × 10^–6^, and λ = 0.015. For further details on the model, see SI.)

To further investigate this hypothesis, relaxation data were obtained for selected samples with varying Cu^II^ concentrations (Figures 5A and S13, Table S4). Increasing the concentration up to 2.5 mM (20 molar equivalents), the echo decay became faster, as would be expected for increasing close-range interactions between Cu^II^ sites. However, data clearly revealed a very marked step upon further increasing the ratio from 20 to 50 molar equivalents of Cu^II^ but keeping the Cu^II^ concentration constant. The resulting echo decay displayed an additional slow component. This slow component completely dominated the remaining signal at even higher equivalents.

Considering that hyperfine spectroscopy is strongly impacted by fast relaxation, these findings explained our observations from EDNMR and ESEEM/HYSCORE, where the substantial changes seen at above 20 molar equivalents of Cu^II^ could not be explained by a mere addition to the spectral signatures visible at lower concentrations. Thus, the slower relaxing Cu^II^ sites dominated the hyperfine spectra in excellent agreement with the relaxation data.

PDS distance measurements (Figures 5B, C and S14–S17, Tables S5–S6) further revealed an almost linear increase in the modulation depth (Δ) with Cu^II^ loading from 1 to ∼12 molar equivalents and plateauing thereafter (up to 20 molar equivalents of Cu^II^). In agreement with the above hypothesis, there was a marked change for the higher-equivalent Cu^II^ samples, with virtually no modulation depth observed from 50 molar equivalents of Cu^II^ (Figure S16). Thus, PDS results were in excellent agreement with the hyperfine experiments, demonstrating once more substantial changes in the spectral signature beyond 20 molar equivalents of Cu^II^, which completely dominated PDS traces and eliminated any modulation depth. We concluded that the high-affinity sites relax fast, while lower-affinity sites that were occupied at higher Cu^II^ loading relax slower. Together, this showed why a simple “additive” assumption to simulate spectra with increasing Cu^II^ loading was not feasible.

Instead, a speciation model was developed assuming two sets of binding sites with different dissociation constants^68^ to mimic high- and low-affinity binding sites, allowing the simulation of PDS modulation depths of HRG pseudo-titration samples in dependence of the Cu^II^ loading (for a full description of the model and simulation parameters, see SI and Wort et al., manuscript in preparation).^50^ Importantly, the model unambiguously demonstrated that experimental modulation depths could be simulated with 12 high-affinity binding sites (*K*_D_ ≪ 1.25 × 10^–4^), while observed plateauing of PDS data could not be satisfactorily described by the model if more high-affinity sites were assumed (Figures 5C and S18–S19, Tables S7–S8). Note, however, that the model did not allow firm conclusions regarding the number or dissociation constant of low-affinity binding sites. It should be emphasized that while PDS (Figure S17) yielded very broad distance distributions that we refrained from quantifying and that did not change significantly within confidence intervals between 1 and 20 equivalents of Cu^II^ added, reliable modulation depth quantitation has been possible and facilitated robust spin counting, as recently benchmarked.^48^

In summary, the presented ITC and EPR data consistently demonstrated that HRG has 10–12 metal ion binding sites of equal and high affinity, which are presumably located in its HRR. Interestingly, a mass spectrometry study on a 35-residue anti-angiogenic HRG peptide derived from the HRR concluded that Zn^II^ binding involves various locations within this region, rather than one single preferred site,^69^ thus strongly supporting the hypothesis of different binding sites in the HRR being randomly occupied. Results from hyperfine spectroscopy strongly suggested the presence of two or more histidine residues coordinating the metal ion per binding site up to 10–15 molar equivalents of Cu^II^, with spectroscopic profiles indicating that all high-affinity sites were of similar geometry and populated statistically. Furthermore, EPR data of high-equivalent Cu^II^ samples (50 or more molar equivalents) suggested that once the higher-affinity sites were occupied, additional metal ion binding occurred at lower-affinity sites that did not involve histidine residues. These data are consistent with the presence of 25 histidine residues within the HRR, which could offer 2 residues per metal ion for up to 12 ions bound. The majority of the HRR histidine residues in HRG are part of a series of tandem repeats of the consensus sequence G[H/P][H/P]PH (see Figure 1).^5,20^ In rabbit HRG, a further 18 histidine residues are spread over the HRR flanking regions including PRR1 and PRR2, bringing the total number to 43 histidine residues within the disordered PRR1-HRR-PRR2 stretch. This is in line with the observation that beyond 50 molar equivalents of Cu^II^, lower-affinity non-histidine binding sites were occupied. Data further indicated that fewer histidine residues were available per metal ion at higher loading, suggesting a variable, adapting fold of the predicted intrinsically disordered PRR1-HRR-PRR2 region. We hypothesize that this arrangement is key for enabling tight regulation of the metal ion concentration in mammalian plasma and that the induced transient structural features lead to different affinities of HRG for interaction partners such as heparin, thus regulating and targeting HRG’s function. Transient free Zn^II^ concentrations in plasma at specific sites (for example, at the surface of activated platelets) can reach very high levels locally; while it is difficult to determine exact concentrations in such transient events, it can be estimated that a molar ratio ≥30 relative to the HRG plasma concentration is likely^1,14,15^ and even required to induce certain functionality.^1^ This is in agreement with our findings that additional lower-affinity binding sites were occupied at high (>20 molar equivalents) Cu^II^ loading, suggesting new transient structural features upon occupation of these additional sites having a direct functional effect. One can envision that the flexible, unstructured PRR1-HRR-PRR2 region wraps around an HRG–partner complex, with the subsequent binding of metal ions locking this transient structure in place to stabilize the complex, while the known plasmin cleavage of the HRR could provide a mechanism for targeted release of metal ions.

## Conclusions

In this study, different EPR techniques were applied, supported by computational structure predication, to gain insight into a native protein that is hardly tractable with high-resolution structural biology methods. We conclude that native mammalian HRG acts like a sponge for Cu^II^ (and other divalent metal ions, with Zn^II^ known to play an important regulatory role), using a set of high-affinity binding sites involving histidine coordination of the metal ions and a much larger number of lower-affinity binding sites not involving histidine residues. We further conclude that the predicted disordered PRR1-HRR-PRR2 region of HRG allows for a gradual and flexible adaptation of structural features dependent on the metal ion loading, as identified by EPR studying the general topology of HRG metal ion binding sites and their chemical environment. Further studies are required to characterize these induced HRR structural features in the context of effector binding and local metal ion concentration. Ideally, a recombinant expression system for HRG could be established, allowing specific modifications and isotope labeling.

To the best of our knowledge, this report demonstrates the first application of a combination of complementary CW, pulse dipolar, and hyperfine EPR approaches to count and characterize multiple endogenous metal ion binding sites in a highly complex biological system, the native mammalian protein HRG, that has so far escaped high-resolution structural characterization.

The research data underpinning this publication will be accessible at https://doi.org/10.17630/e4405284-4aa6-43b7-9074-744935ef3ccf.^70^

## Supporting Information Available

Detailed experimental procedures, HRG JPred4 and AF2 structure prediction, CW EPR data, hyperfine spectroscopy and PDS data, and derivation of the speciation model (PDF)
