# Supplementary material for: Investigating Native Metal Ion Binding Sites in Mammalian Histidine-Rich Glycoprotein
Source: J Am Chem Soc. 2023 Mar 31;145(14):8064–72. doi: 10.1021/jacs.3c00587 (PMC10103162; doi:10.1021/jacs.3c00587)
Supplement: Supplementary file 1 — ja3c00587_si_001.pdf [file ja3c00587_si_001.pdf]

Supplementary Information

Investigating Native Metal Ion Binding Sites in Mammalian Histidine-Rich Glycoprotein

Katrin Ackermann,[1,2,3] Siavash Khazaipoul,[2,3,4] Joshua L. Wort,[1,2,3] Amélie I. S. Sobczak,[2,3,4] Hassane El Mkami,[3,5] Alan J. Stewart*[2,3,4] and Bela E. Bode*[1,2,3]

[1]EaStCHEM School of Chemistry, University of St Andrews, North Haugh, St Andrews, KY16 9ST, Scotland; [2]Biomedical Sciences Research Complex, University of St Andrews, North Haugh, St Andrews, KY16 9ST, Scotland; [3]Centre of Magnetic Resonance, University of St Andrews, North Haugh, St Andrews, KY16 9ST, Scotland; [4]School of Medicine, University of St Andrews, North Haugh, St Andrews, KY16 9TF, Scotland; and [5]School of Physics and Astronomy, University of St Andrews, North Haugh, St Andrews, KY16 9SS, Scotland

*E-mail: ajs21@st-andrews.ac.uk

*E-mail: beb2@st-andrews.ac.uk

Table of contents

1) Experimental procedures Page 2
2) JPred4 and AlphaFold2 structure prediction Page 7
3) Continuous wave EPR data Page 9
4) Hyperfine spectroscopy Page 12
5) Pulse dipolar EPR Page 25
6) Speciation model to simulate PELDOR modulation depths in HRG Page 30
7) References Page 34

1) Experimental procedures

Protein purification

HRG was purified from rabbit serum (Sigma Aldrich, Poole UK) using immobilised Ni^{2+}-affinity chromatography (HisTrap column; Cytiva, Little Chalfont, UK). Prior to purification the serum was centrifuged at 4,000 × g for 30 min at 4°C, filtered through a 0.45 µm filter (Sartorius, Epsom, UK) and imidazole was added to a final concentration of 5 mM. The resultant fractions containing HRG were further subjected to anion exchange (HiTrap DEAE FF column, Cytiva) and gel filtration (HiLoad Superdex-75 column, Cytiva) chromatography. All purification steps were carried out using an ÄKTA Purifier (Cytiva). Prior to any experiment, the purified protein was dialysed in the appropriate buffer.

Isothermal titration calorimetry

Isothermal titration calorimetry (ITC) was performed using a MicroCal iTC200 instrument (Cytiva). Prior to titration, rabbit HRG and $CuCl_2$ (Sigma Aldrich, Poole UK) were dissolved separately in ITC buffer (140 mM NaCl, 50 mM Tris, pH 7.4) to final concentrations of 10 µM and 1.5 mM, respectively, at 25°C and pH was re-adjusted to 7.4 to avoid mismatch conditions. The titration involved a single injection of 0.4 µl of $CuCl_2$ over 0.8 s followed by 18 injections of 2 µl $CuCl_2$ over 5 s with a 150 s interval between injections to allow full equilibration. The stirring speed was set to 750 rpm throughout. Additionally, a control experiment was carried out whereby the titrant was injected into buffer and the resultant heats were deducted from those obtained in the main experiment.

Heparin binding assay

Zinc ion binding to HRG is known to increase the affinity of this protein to unfractionated heparin (UFH). In order to assess whether Cu^{II} ions elicit a similar effect, binding of HRG to immobilised UFH in the presence and absence of Zn^{II} and Cu^{II} was performed. For this Costar Carbohydrate Binding Plates (Sigma-Aldrich, Poole, UK) were used. UFH (Acros Organics, Loughborough, UK) was activated with 15 mM sodium periodate according to the manufacturer's protocol. Purified rabbit HRG (rHRG) was biotinylated using a Biotinylation Kit (Stratech, Newmarket, UK) and then dialysed overnight at room temperature against 50 mM HEPES, 150 mM NaCl, 0.2 % Tween 20, pH 7.4. The activated UFH was dialysed twice for 1 h at room temperature against 0.1 M sodium acetate, pH 5.5. The activated UFH (10 µg/well) was then bound to the plate overnight. Wells were washed with 50 mM HEPES, 150 mM NaCl, 0.2 % Tween 20, pH 7.4 and then blocked with 50 mM Tris buffer pH 8.2, 0.2 % fish gelatine for 1 h at room temperature. Biotinylated rHRG was added to the wells (0-3 µM) for 2 h at 37 °C in the absence and presence of 50 µM $ZnCl_2$ or 50 µM $CuCl_2$. After washing with 50 mM HEPES, 150 mM NaCl, 0.2 % Tween 20, pH 7.4, binding was detected with alkaline phosphatase-linked streptavidin (1:10,000

dilution, ThermoFisher Scientific, Loughborough, UK) and *p*-nitrophenol phosphate substrate (200 µg/well, Sigma-Aldrich). The reaction was stopped with 3 M NaOH and the absorbance was read at 405 nm using a Dynex MRX spectrophotometer (Dynex Technologies, West Sussex, UK).

Electron Paramagnetic Resonance (EPR) spectroscopy

EPR sample preparation

Pseudo-titration samples were prepared with a protein concentration of 250 μM in buffer containing 50 mM Tris and 140 mM NaCl at pH 7.4 and Cu^{II} from 1 to 20 molar equivalents (from 250 μM to 5.0 mM). The control sample contained no protein and 250 μM Cu^{II}. Two batches of protein were prepared, the first one yielded the control sample and pseudo-titration samples from 1 to 10 molar equivalents of Cu^{II}; the second batch yielded pseudo-titration samples at 5 and 10 molar equivalents Cu^{II} (repeat samples for reproducibility), and the 12, 15, and 20 molar equivalent samples. Samples were mixed with equal amounts of ethylene glycol for cryoprotection, resulting in a final protein concentration of 125 μM and Cu^{II} concentration of 125 μM to 2.5 mM, transferred to 3 mm (70 μL sample volume) quartz EPR tubes, and immediately frozen in liquid nitrogen.

To determine the maximum binding capacity of HRG four further samples were prepared at a final Cu^{II} concentration of 2.5 mM, thus corresponding to the Cu^{II} concentration in the 20 molar equivalents sample of the pseudo-titration. Samples had final protein concentrations of 50, 25, 12.5, and 6.25 μM, corresponding to 50, 100, 200, and 400 molar equivalents of added Cu^{II}, respectively.

Note that Tris buffer shows weak complexation of free copper ions, leading to ^{14}N hyperfine interactions. However, other commonly used buffers such as PBS buffer, lead to precipitation resulting in reduced Cu^{II}-loading.[1] Indeed, there are very few (if any) options for buffers that do not complex free copper ions. This is highlighted by a review on the use of pH buffers and their interaction with metal ions, where all of the buffers examined (out of the 31 buffers listed) exhibited the capacity to undergo complexation with copper ions.[2] Therefore, for consistency and comparability ITC buffer (140 mM NaCl, 50 mM Tris, pH 7.4, see above) was used for EPR sample preparation.

Continuous wave (CW) EPR

CW EPR spectra were obtained at 120 K with a Bruker EMX 10/12 spectrometer running Xenon software and equipped with an ELEXSYS Super Hi-Q resonator at an operating frequency of ~9.5 GHz with 100 kHz modulation. Temperature was controlled with an ER4141 VTM Nitrogen VT unit (Bruker) operated with liquid nitrogen. CW spectra were recorded using a 160 mT field sweep centred at

310 mT, a time constant of 40.96 ms, a conversion time of 6.67 ms, and 8000 points resolution. An attenuation of 10.0 dB (20 mW power) and a modulation amplitude of 0.2 mT were used. CW spectra were phase- and background-corrected and the double integral was obtained using the Xenon software. Spectra were field-corrected using DPPH as a standard.

Pulse experiments

Pulse experiments were performed at X- (9.5 GHz) and at Q-band (34 GHz) both operating on a Bruker ELEXSYS E580 spectrometer, with probe-heads supporting a split ring resonator (4118X-MS3) for X-band and a 3 mm cylindrical resonator (ER 5106QT-2w in TE012 mode) for Q-band, respectively. Pulses were amplified by pulse travelling wave tube (TWT) amplifiers (Applied Systems Engineering) with nominal output of 1 kW and 150 W at X- and Q-band, respectively. Temperature was controlled via cryogen free variable temperature cryostats (Cryogenic Ltd) operating in the 3.5-300 K temperature range.

Pulse dipolar EPR

Pulsed electron-electron double resonance (PELDOR or DEER) measurements were recorded at Q-band frequencies (34 GHz) using a second frequency option (E580-400U).

Temperature optimisation for PELDOR experiments were performed between 10 K and 50 K using the HRG sample with 2 equivalents of Cu^{II}. Here, T_2 (or T_m) was determined from a 2-pulse decay experiment with stretched exponential decay for fitting, and T_1 was the determined longer τ from an inversion recovery experiment. Determined relaxation times were then used to assess the relative sensitivity per temperature as described previously.[3] T_2 experiments were further performed on selected samples to investigate the change in relaxation behaviour between 1 and 400 molar equivalents of Cu^{II}.

PELDOR experiments were performed using the four-pulse DEER[4-6] pulse sequence $\pi/2(\nu_A) - \tau_1 - \pi(\nu_A) - (\tau_1 + t) - \pi(\nu_B) - (\tau_2 - t) - \pi(\nu_A) - \tau_2$ – echo at 30 K as described previously,[7] with a frequency offset (pump – detection frequency) of +80 MHz (~3 mT). Shot repetition times were set to 300 μs; τ_1 was set to 380 ns, and τ_2 to 3300 ns. Pulse lengths used were 16 and 32 ns for $\pi/2$ and π detection, and 14 ns for the ELDOR π pump pulse. The pump pulse was placed on the resonance frequency of the resonator and ~1.7 mT away from the maximum of the spectrum to lower field to allow placing pump and detection positions symmetrically around the maximum.

PELDOR data were analysed using DeerAnalysis2015.[8] Raw experimental PELDOR data were cut at 2500 ns to remove artefacts at the end of the time trace, and background-corrected using a mono-exponential decay function with the background start point set to 1250 ns before subjecting the trace to Tikhonov regularisation. A regularization parameter α of 100,000 was chosen by visual inspection for all traces. Further statistical analysis of PELDOR data was performed as described previously,[9] using the validation tool of DeerAnalysis 2015.[8] Briefly, the background start time was varied from 5% to 80% of the dipolar evolution time in 16 trials and 50% random noise was added (level 1.50) with 50 trials, yielding 800 trials per trace. Trials were pruned, keeping only those where the root mean square deviation (rmsd) was within 15% of the rmsd of the best fit; these trials were further used for determination of the mean modulation depth and error.

Hyperfine spectroscopy

3-pulse electron spin echo envelope modulation (ESEEM)[10-12] spectroscopy was performed at 30 K at X-band frequencies (~9.5 GHz) and on the maximum of the field-swept spectrum with a pulse length of 16 ns for $\pi/2$ detection and inter-pulse delay τ set at the blind spot of the proton (~216 ns). The delay T, set at 280 - 300 ns, was incremented with a dwell time ΔT of 8 ns and a 4-step phase cycle was used. Four dimensions were recorded, whereby the inter-pulse delay was incremented by 0.5 τ_1(H); the first τ was selected for further processing and setting up the HYSCORE experiment (see below). Data were analysed by fitting an exponential decay background function to the (phase-corrected) raw data, subtracting the raw data by this background function, and then dividing the difference by the background function, thus retaining amplitude information after fast Fourier transformation (FFT) similar as described previously.[13] The resulting trace was further subjected to a Hamming window, zero-filling and FFT, before obtaining the absolute (or magnitude) spectrum.

Hyperfine sublevel correlation (HYSCORE)[14] spectroscopy was performed at 15 K at X-band frequencies (~9.5 GHz) and on the maximum of the field-swept spectrum with a pulse length of 16 and 32 ns for $\pi/2$ and π, respectively, τ set at the blind spot of the proton (~216 ns) as chosen from the 3-pulse ESEEM, $t_1 = t_2 = 56$ ns, and a 4-step phase cycle. Data were processed and analysed using the Matlab plugin HYSCOREAN,[15] employing Hamming apodization, zero-filling, 3rd order polynomial background correction, and diagonal and anti-diagonal spectral symmetrisation, keeping a similar amount of noise for each spectrum by adjusting the minimum contour level percentage accordingly.

EDNMR[16-17] measurements were conducted by using an additional second frequency option (E580-400U) jointly with the Q-band setup described above at 30 K. Measurements were taken at low field

(1.0240 – 1.0630 T) and high field (1.1674 – 1.1833 T) on a Hahn echo with an ELDOR HTA (high turning angle) pulse length of 8 µs and an ELDOR attenuation of 30 dB. Only low-field spectra are shown as they offer increased resolution. Note that directly coordinated nitrogen atoms visible in EDNMR would not contribute to the ESEEM signal under our experimental conditions (pulse lengths of $\pi/2$ = 16 ns at X-band).

Simulations

Representative numerical simulations of 3-pulse ESEEM data were performed with EasySpin,[18] using the implemented simulation function "saffron". Prior to simulation, data was background corrected, apodised with a Hamming window, zero-filled and Fourier transformed. The 3-pulse ESEEM simulations were conducted in the frequency domain. EDNMR simulations for the control and HRG with 1 equivalent of Cu^{II} were performed as described previously.[19] The central blind spot was modelled by a sum of Gaussian and Lorentzian lines and subtracted from both raw data sets.

2) Structure prediction

JPred4 secondary structure prediction

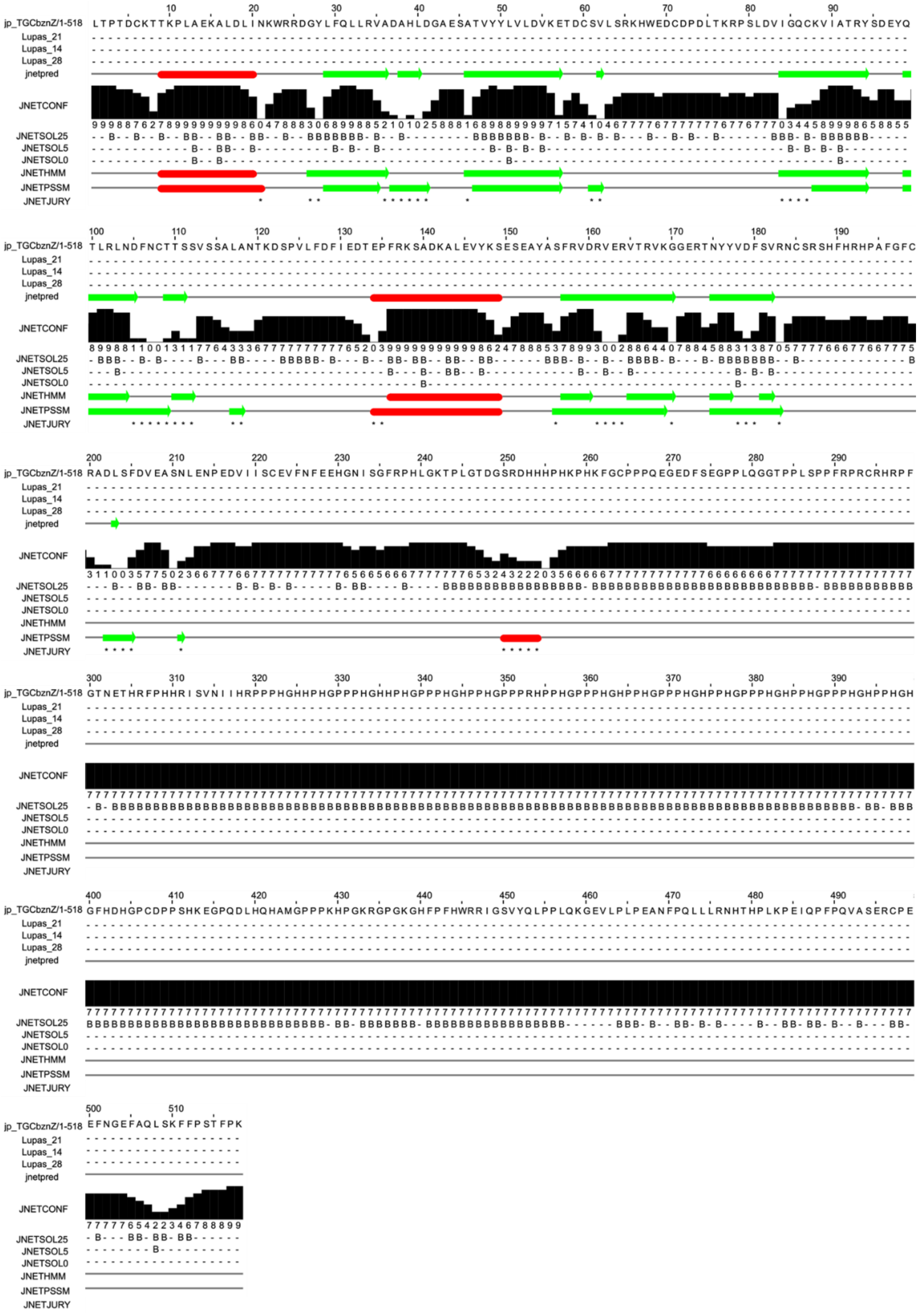

Figure S1: JPred4 secondary structure prediction for rabbit HRG (UniProt accession number Q28640). JNet version: 2.3.1; UniRef90 release: 2014_07, 09-Jul-2014.

AlphaFold2 (AF2) structure prediction

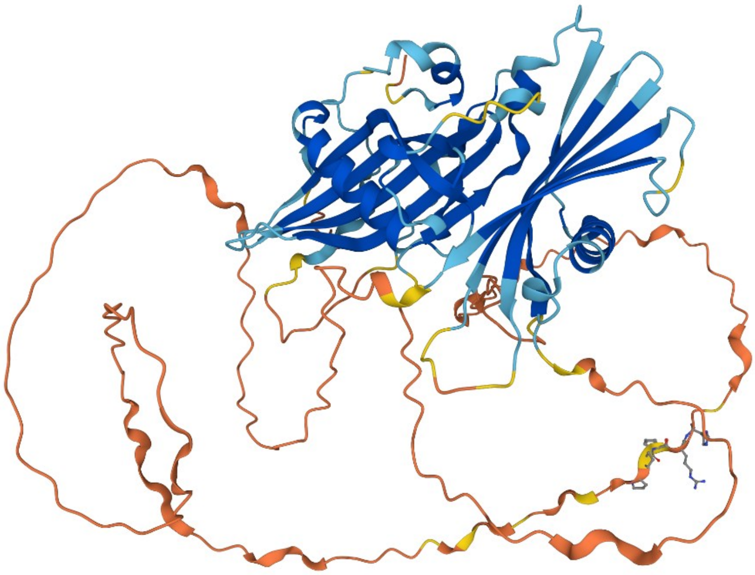

Figure S2: AF2 prediction for rabbit HRG (UniProt Q28640). Colours indicate pLDDT confidence scores between 0 and 100; very low pLDDT scores (below 50 = orange colour) are likely to be unstructured or intrinsically disordered; note that the prediction does not inform on the relative likelihood of different conformations of such regions.

3) Continuous wave (CW) EPR data

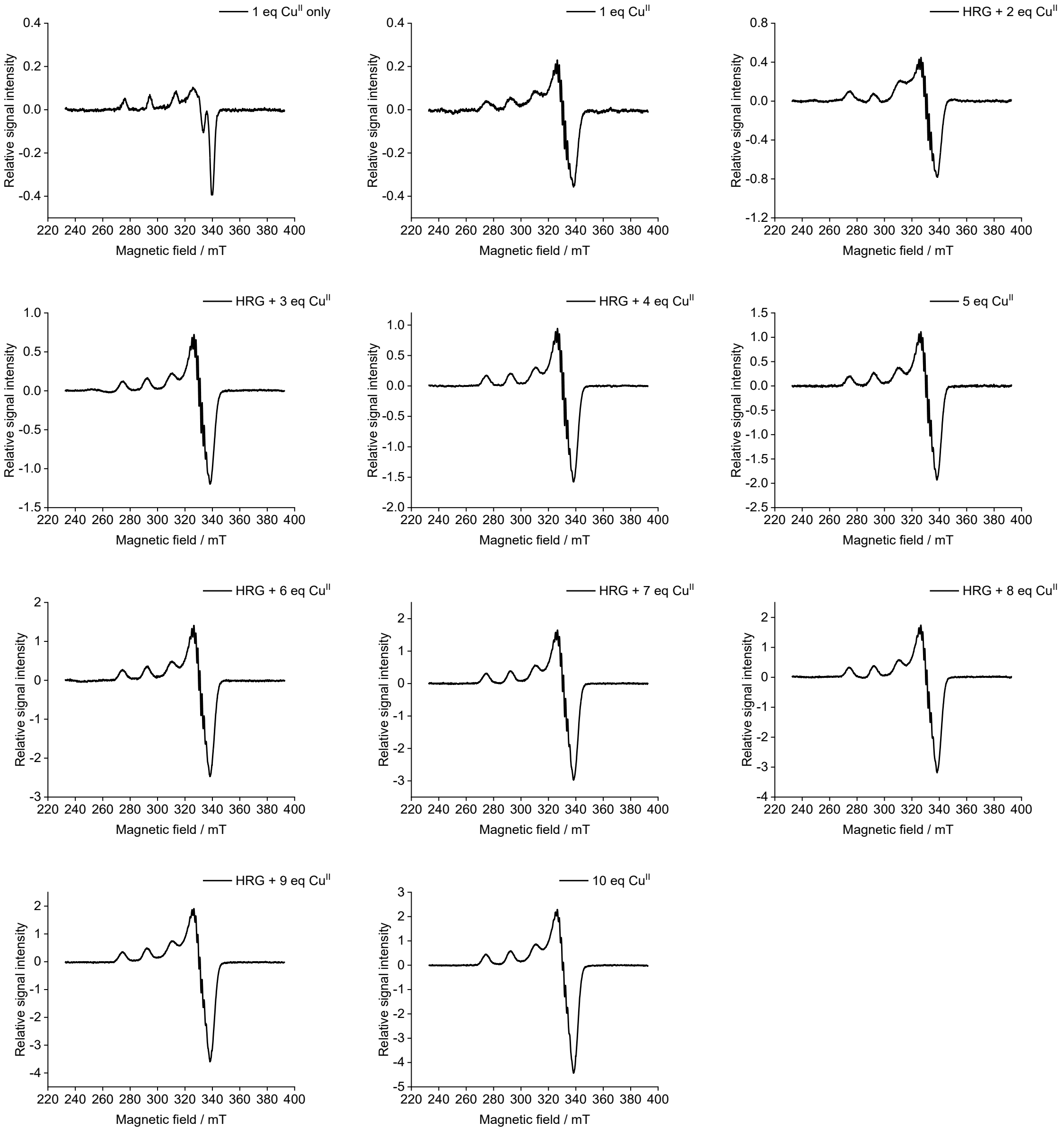

Figure S3. Continuous wave (CW) EPR spectra. Shown are the individual CW EPR spectra obtained for the first batch of the pseudo-titration series consisting of 1 to 10 molar equivalents of Cu^{II} and control sample.

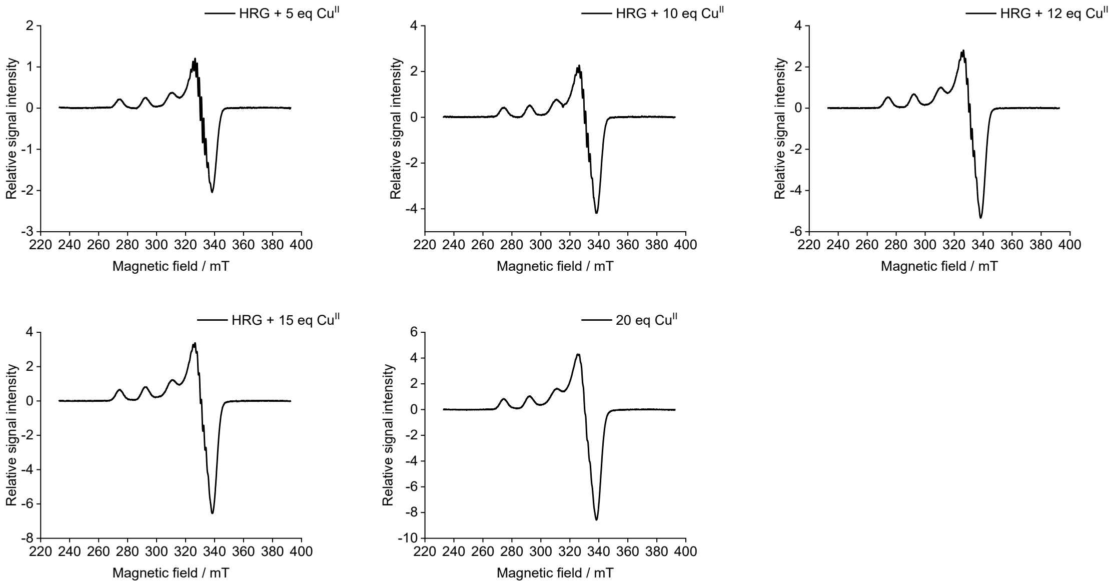

Figure S4. Continuous wave (CW) EPR spectra. Shown are the individual CW EPR spectra obtained for the second batch of the pseudo-titration series consisting of the samples with 5, 10 (both biological repeats) and > 10 molar equivalents of Cu^{II}.

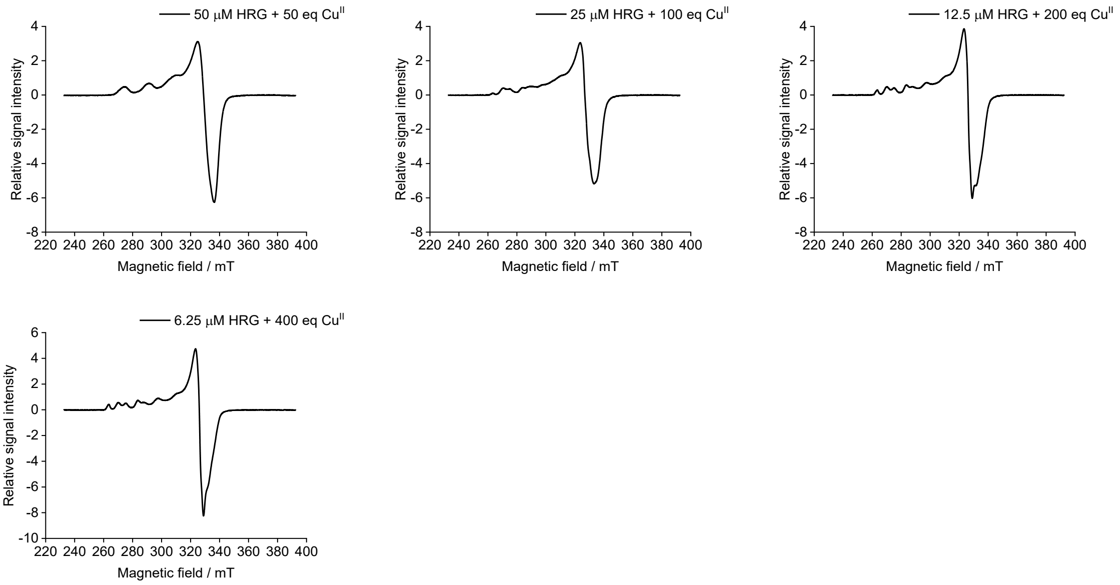

Figure S5. Continuous wave (CW) EPR spectra. Shown are the individual CW EPR spectra obtained for the high equivalent samples (50, 100, 200, and 400 equivalents of Cu^{II}).

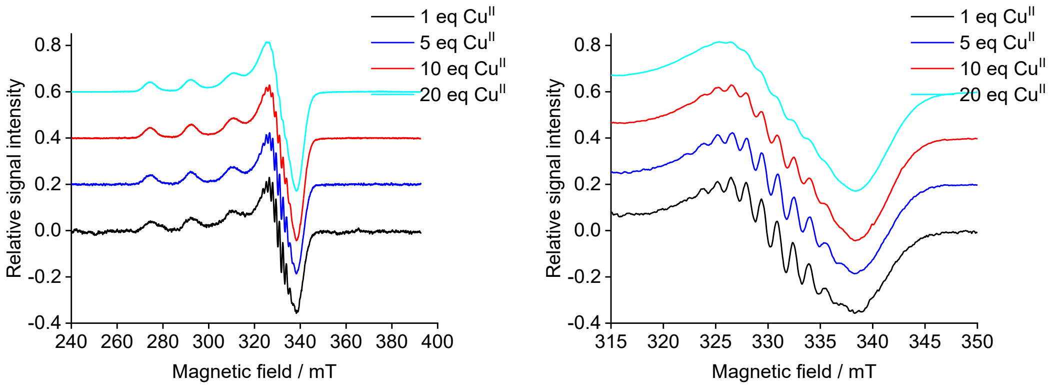

Figure S6. Stacked overlays of CW EPR spectra for comparison of four molar equivalents (1, 5, 10, and 20) of Cu^{II} to illustrate the gradual decrease of SHF splittings. The right plot is zoomed into the SHF region for better visualisation.

Decreasing resolution of SHF upon addition of Cu^{II} was in agreement with earlier data.[20] While this could in principle be caused either by broadening from introduction of dipolarly coupled close-by Cu^{II} or by (un-)specific binding of Cu^{II} to non-histidine sites, the latter is unlikely as the resolution of the signal was lost more quickly from 10 molar equivalents of Cu^{II} onwards than a mere dilution of one type of sites would explain. Thus, either there is dipolar broadening or existing binding sites change. Considering an additive spectrum, where an additional component was added to the existing spectrum exhibiting ^{14}N SHF couplings, one could not explain our observations. Here, while the amplitude of the resolved SHF couplings seemed roughly halved going from 5 to 10 molar equivalents of Cu^{II}, instead of being halved again going from 10 to 20 equivalents they have almost completely vanished at this Cu^{II} loading. This suggested that the situation was more complicated and that spectra could not be simulated simply by assuming 10 binding sites with SHF and 10 binding sites without. Instead, there was either spectral broadening involved or SHF of initial binding sites were changing upon occupation of additional sites. This situation rendered simulations based on simply adding new species with added Cu^{II} unfeasible.

**Compound**	**A_{II} / mT**	**g_{II}**
Cu^{II} in water (single species)	12.34	2.420
Cu^{II} in Tris buffer (single species)	18.20	2.223
Main species in HRG + 5 eq. Cu^{II}	17.93	2.251
New species in HRG + 200 eq. Cu^{II}	13.59	2.336

Table S1. Measured values for A_{II} and g_{II}. DPPH was used as a reference.

4) Hyperfine spectroscopy

EDNMR

Effect of buffer coordination: The control sample containing no protein showed peaks arising from the interaction of Cu^{II} with nitrogen atoms of the Tris buffer (Figure S7).

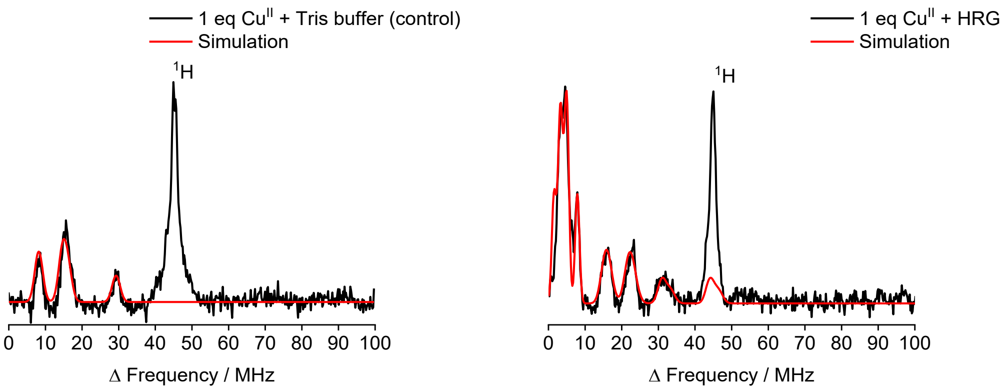

Figure S7. EDNMR simulations for the control (Cu^{II} in Tris buffer; left) and HRG with 1 molar equivalent of Cu^{II} (right). Only the right-hand side of the EDNMR spectra are shown and the central blind spot has been subtracted from both raw data sets. Parameters to simulate the EDNMR spectrum of Cu^{II} in Tris buffer are given in Table S2.

A good agreement between experiment and simulation of both the control EDNMR spectrum and the spectrum in presence of HRG could be obtained, not considering the peak of the proton Larmor frequency (as indicated in Figure S7). Details of hyperfine parameters used for the simulations are given in Table S2 below. No further simulations considering different binding sites were performed (see below for discussion).

	$g_\parallel$	$A_\parallel$ [a,b]	A_1 [a,c]	A_2 [a,c]	A_3 [a,c]	A_1 [a,d]	A_2 [a,d]	A_3 [a,d]
Cu^{II}-Tris	2.233 ± 0.005	566 ± 4	24 ± 1	24 ± 1	23 ± 1			
Cu^{II}-HRG	2.274 ± 0.005	562 ± 4	40 ± 1	40 ± 1	41 ± 1	39 ± 1	39 ± 1	38 ± 1

Table S2. EPR parameters of Cu^{II} and super-hyperfine couplings of nitrogen atoms belonging to Tris-buffer and two inequivalent imino nitrogen nuclei of histidine imidazole rings. [a] *A* values are given in MHz ± 1. [b] $A_\parallel$ is given for Cu^{II}. [c] $A_{1,2,3}$ are given for N_1; [d] $A_{1,2,3}$ are given for N_2.

In the presence of HRG, EDNMR spectra showed additional peaks with one coinciding with the proton Larmor frequency contribution, as well as a broad background feature. Based on the simulations the positions of the defined new peaks confirmed direct coordination of at least two imino nitrogen nuclei of imidazole rings to Cu^{II} with a mainly isotropic hyperfine coupling caused by significant electron spin density delocalisation onto these nuclei that could not be attributed to histidine residues forming the Cu^{II} binding sites.

These hyperfine couplings were apparent already in presence of 1 molar equivalent of Cu^{II}, and EDNMR spectra remained virtually unchanged with increasing Cu^{II} concentration up until 15-20 equivalents. This indicated that, within this metal ion ratio, all populated histidine binding sites showed very similar binding geometries. Speculatively, the broad background feature may be attributed to heterogeneity in coordination of the Cu^{II} to the high affinity binding sites, however it is important to note that no other specific couplings (which would indicate specific sites) were resolved. Further increasing the relative amount of Cu^{II} (50 and more molar equivalents of Cu^{II}) led to the loss of the histidine-associated peaks, suggesting that non-histidine binding sites became dominant. In agreement with the SHF observed in the CW EPR spectra, the histidine-associated peaks have been lost at 50 molar equivalents of Cu^{II} and above. If one assumed a mere additive behaviour for the composition of the spectrum, then 40% of histidine-associated peaks should remain visible at 50 molar equivalents of Cu^{II}.

These data led to the hypothesis that different metal ion binding sites have different relaxation behaviour, where Cu^{II} bound to high-affinity sites relaxes faster and is thus contributing significantly less to echo detected experiments once lower-affinity sites are being occupied, as otherwise one would expect the hyperfine coupling to be recoverable even from the broadened spectra.

3-pulse ESEEM

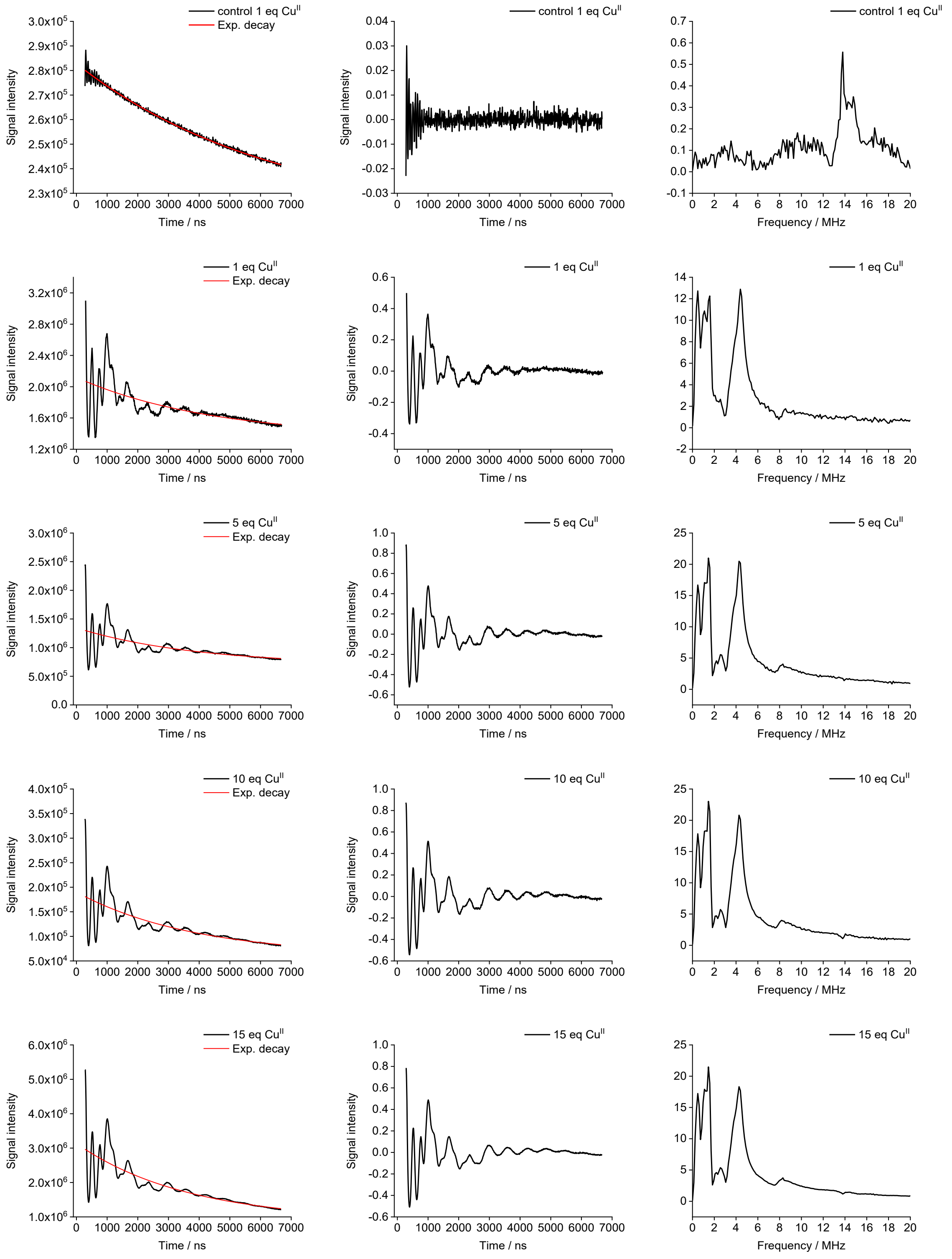

Figure S8. Individual ESEEM spectra of the control and HRG with 1, 5, 10, 15, 20, 50, 100, 200, and 400 molar equivalents of Cu^{II}. Shown are the raw ESEEM traces (black) with fitted exponential background decay (red, left), the background-corrected traces (middle), and corresponding absolute (magnitude) spectra after FFT (right).

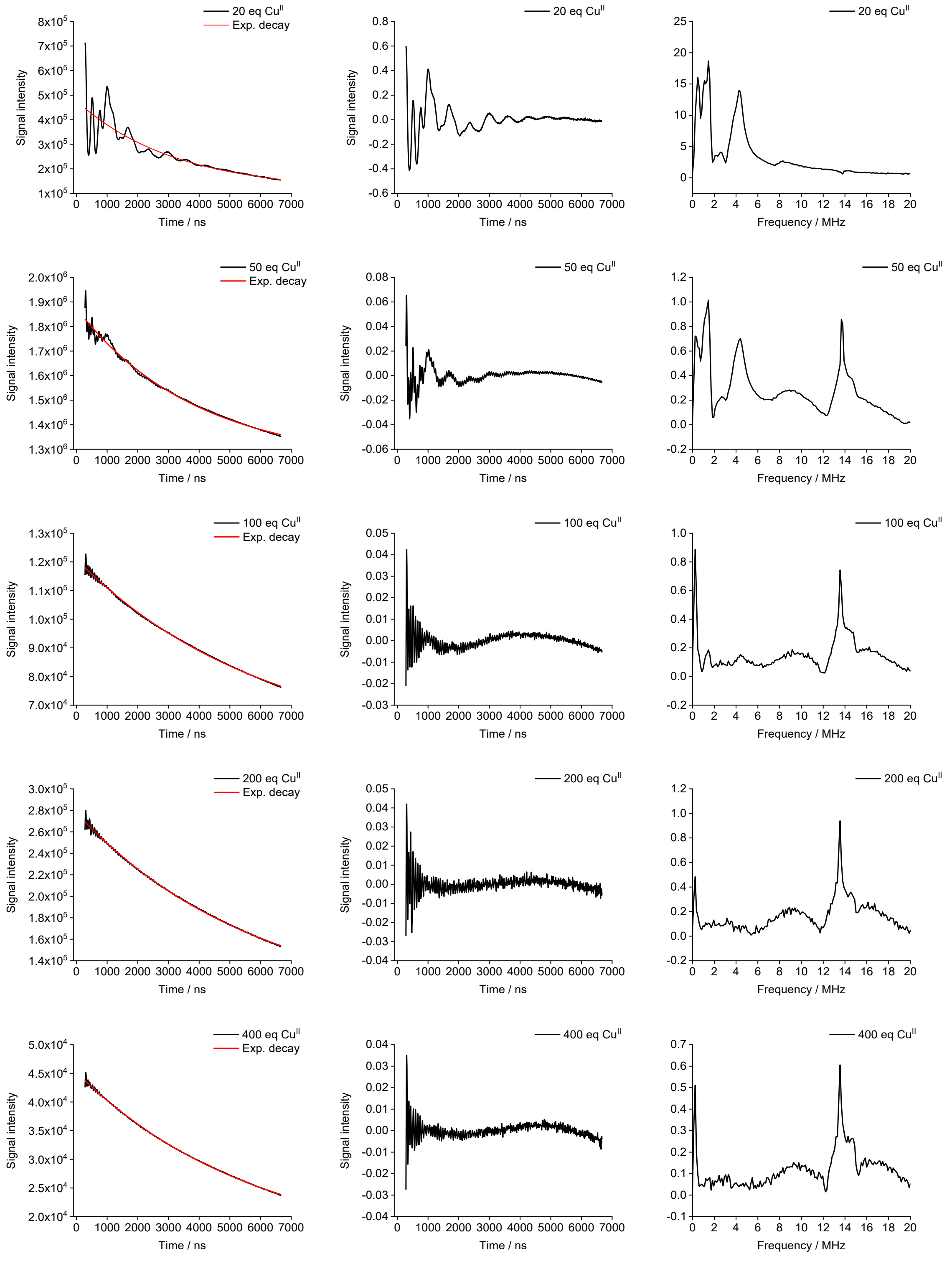

Figure S8, continued. Individual ESEEM spectra of the control and HRG with 1, 5, 10, 15, 20, 50, 100, 200, and 400 molar equivalents of Cu^{II}. Shown are the raw ESEEM traces (black) with fitted exponential background decay (red, left), the background-corrected traces (middle), and corresponding absolute (magnitude) spectra after FFT (right).

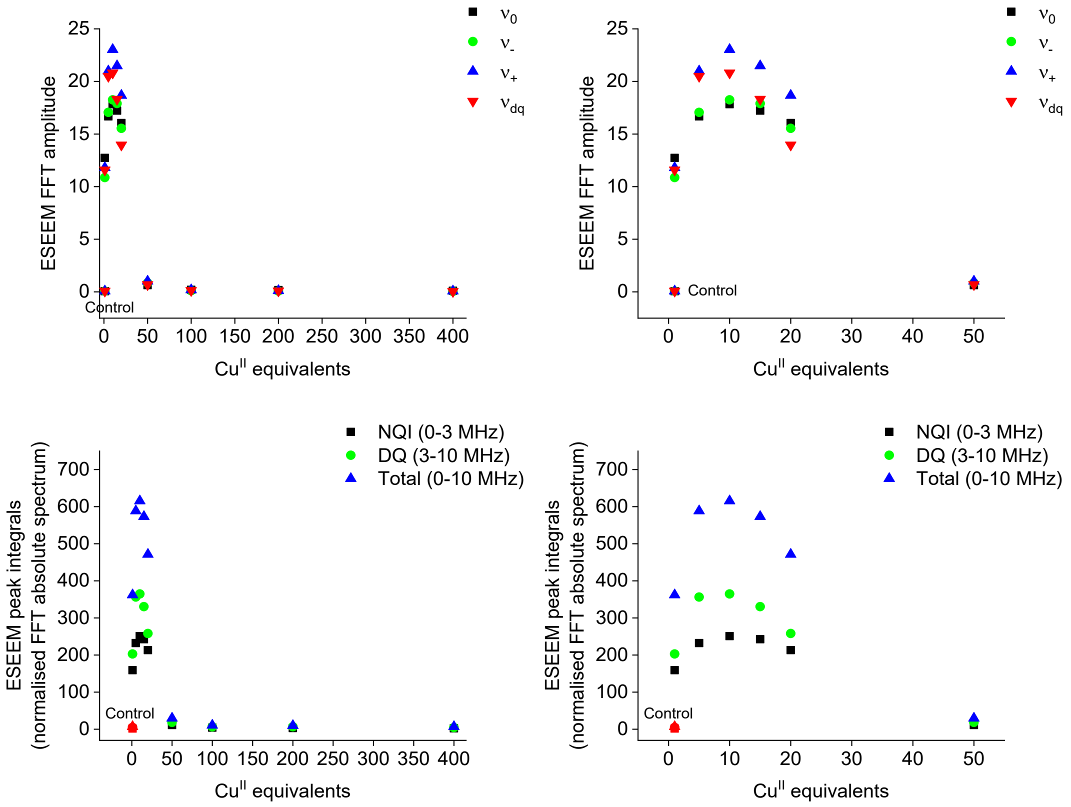

Figure S9. Quantitative analysis of FFT amplitudes and peak integrals from ESEEM spectra for 1 to 400 molar equivalents of Cu^{II}. Top: FFT amplitudes for nuclear quadrupole peaks and double quantum peaks (left) and zoom-in (right). Amplitudes were read out at fixed frequencies for nuclear quadrupole interactions and double quantum transitions: ν_0 at 0.488 MHz, ν_- at 1.099 MHz, ν_+ at 1.465 MHz and ν_{dq} at 4.272 MHz. Bottom: Peak integrals for nuclear quadrupole interactions (NQI; 0-3 MHz), double quantum interactions (DQ; 3-10 MHz), and total integrals (0-10 MHz) are shown on the left, with zoom-in on the right.

Quantitative analysis of ESEEM peak amplitudes after Fourier transformation (FFT) and peak integration provided further support for our EDNMR data:

At up to 20 molar equivalents of Cu^{II}, the FFT amplitudes were relatively constant, while at higher molar equivalents (≥50) they were substantially reduced. The intensity of the DQ peak (or the DQ peak integral) depends on the number of histidine residues involved in the binding site.[21] DQ peak integral values were fairly stable between 5 to 15 molar equivalents of Cu^{II} but decreased at 20 molar equivalents, suggesting that less histidine residues per Cu^{II} were available for binding from this point. At up to 15 molar equivalents, quantitation of the DQ peak suggested coordination of the Cu^{II} by at least two imidazole rings, which was also in good agreement with simulations (see below).[13, 21] At 1 molar equivalent of Cu^{II} NQI and DQ peak integrals were slightly lower than between 5 to 15, again

suggesting binding to less than two histidine residues; the reason for this is not clear but might be due to some competition with the Tris buffer.

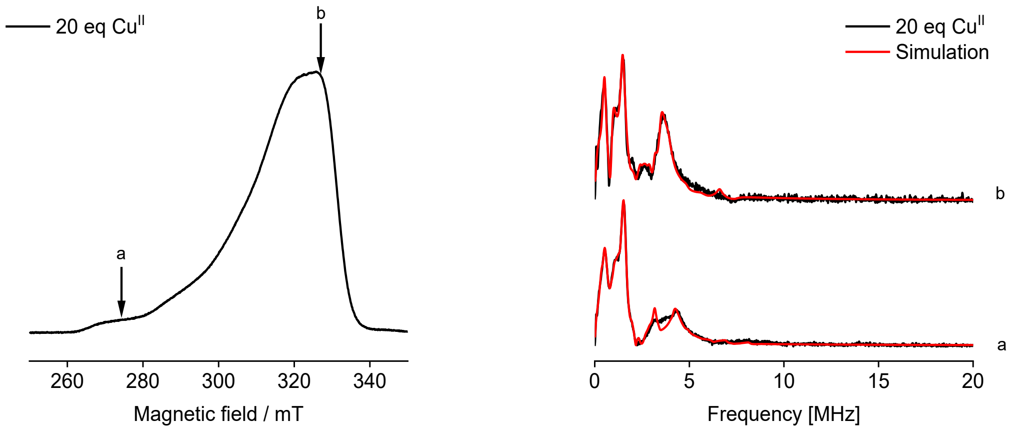

Figure S10. Field-swept spectrum (left) with arrows indicating the field positions for the ESEEM spectra shown on the right for HRG with 20 molar equivalents of Cu^{II}, with simulations (red) demonstrating a good fit to experimental data (black) could be obtained assuming two remote histidine nitrogen atoms (see Table S3 for simulation parameters).

Parameter[a]	N_{rem1}	N_{rem2}
A_x	2.75 ± 0.05	2.5 ± 0.1
A_y	1.76 ± 0.05	2.05 ± 0.05
A_z	1.17 ± 0.05	1.32 ± 0.05
A_{iso}	1.9 ± 0.1	2.0 ± 0.1
$\|A_{dip}\|$	0.9 ± 0.1	0.5 ± 0.1
e^2qQ/h	1.55 ± 0.05	1.5 ± 0.1
η	0.65 ± 0.05	0.75 ± 0.05

Table S3. Hyperfine and quadrupolar parameters of two remote histidine nitrogen (N_{rem}) atoms derived from the simulation of three-pulse ESEEM data. [a] All parameters except η are given in MHz.

HYSCORE

HYSCORE with 1 molar equivalent of Cu^{II}

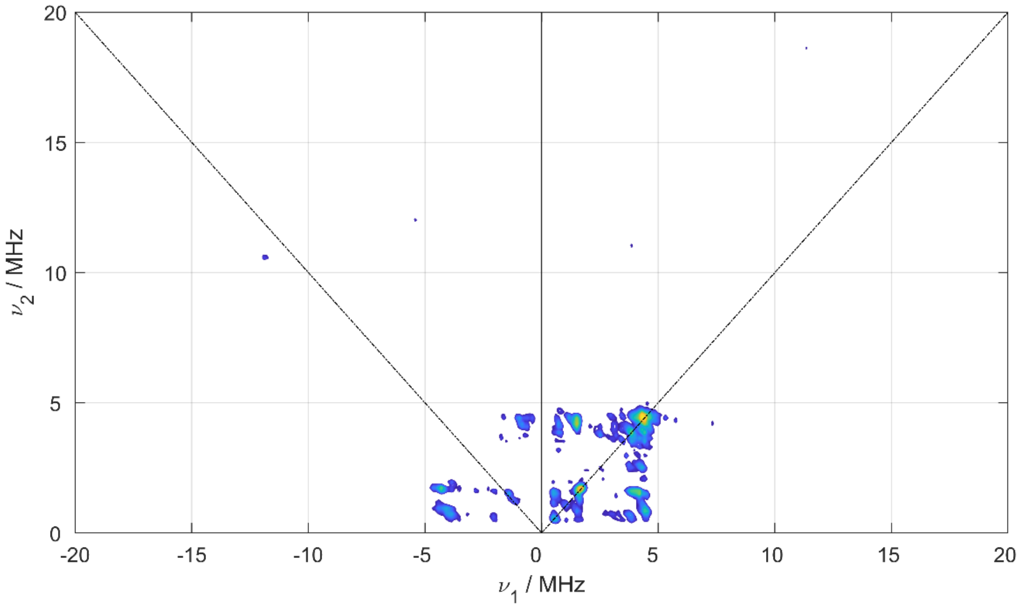

HYSCORE with 5 molar equivalents of Cu^{II}

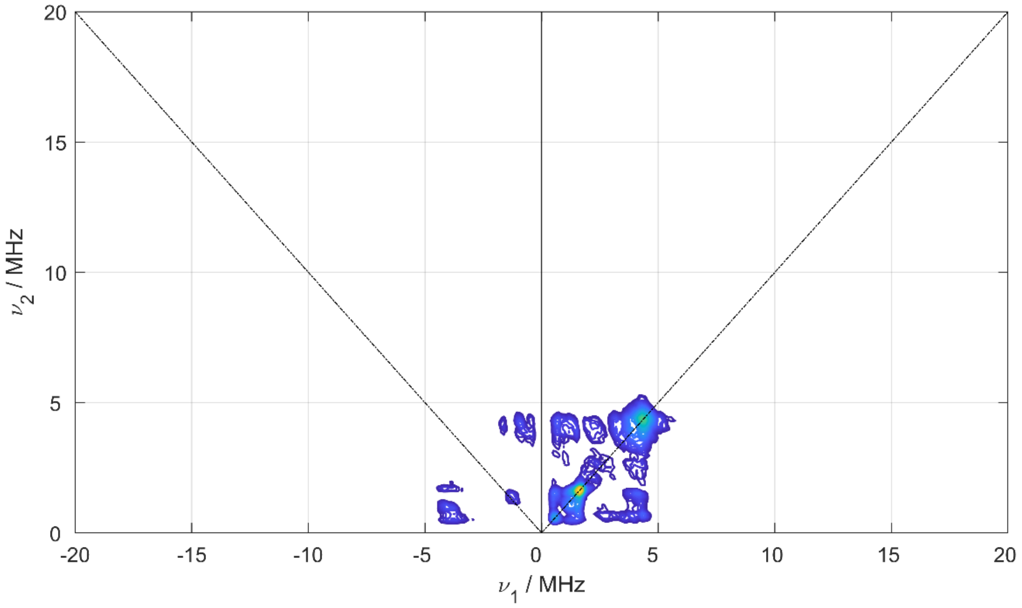

Figure S11. Individual HYSCORE spectra. From top to bottom: 1 / 5 / 10 / 15 / 20 / 50 / 100 / 200 / 400 equivalents Cu^{II} and control (Cu^{II} in Tris buffer). See also Figure 4C in the manuscript.

HYSCORE with 10 molar equivalents of Cu^{II}

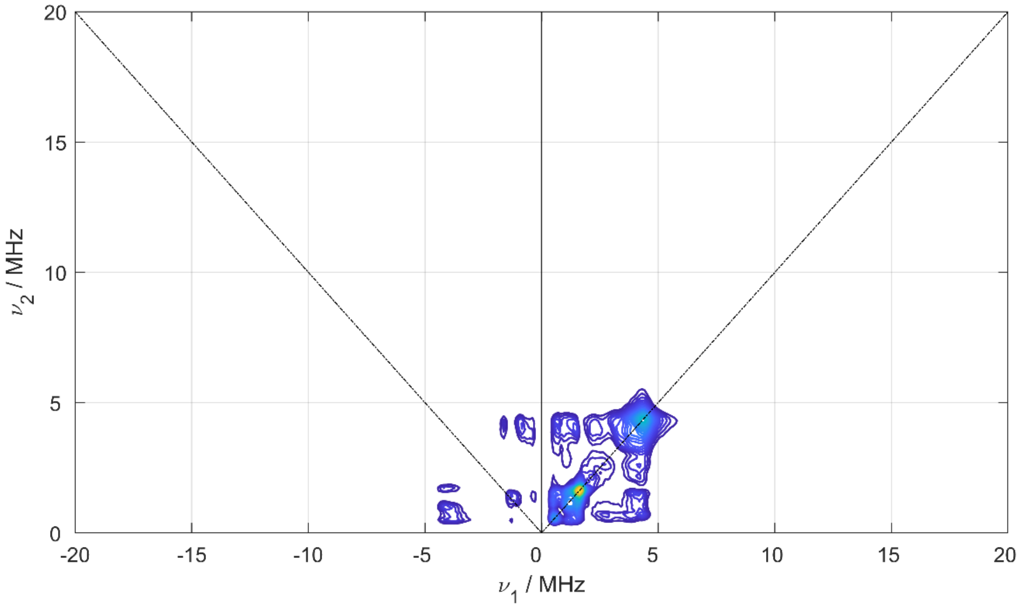

HYSCORE with 15 molar equivalents of Cu^{II}

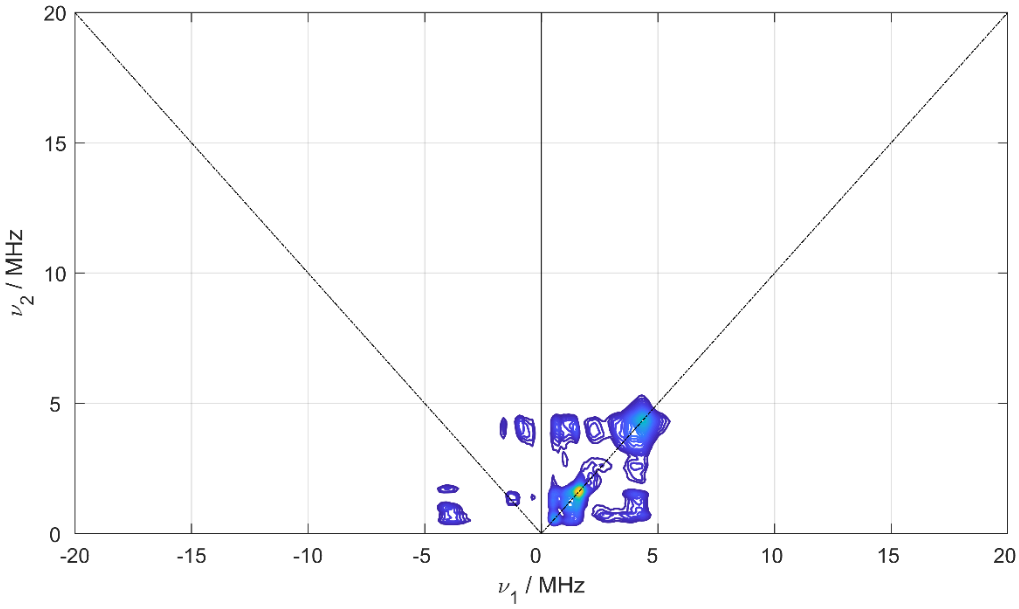

Figure S11, continued. Individual HYSCORE spectra. From top to bottom: 1 / 5 / 10 / 15 / 20 / 50 / 100 / 200 / 400 equivalents Cu^{II} and control (Cu^{II} in Tris buffer). See also Figure 4C in the manuscript.

HYSCORE with 20 molar equivalents of Cu^{II}

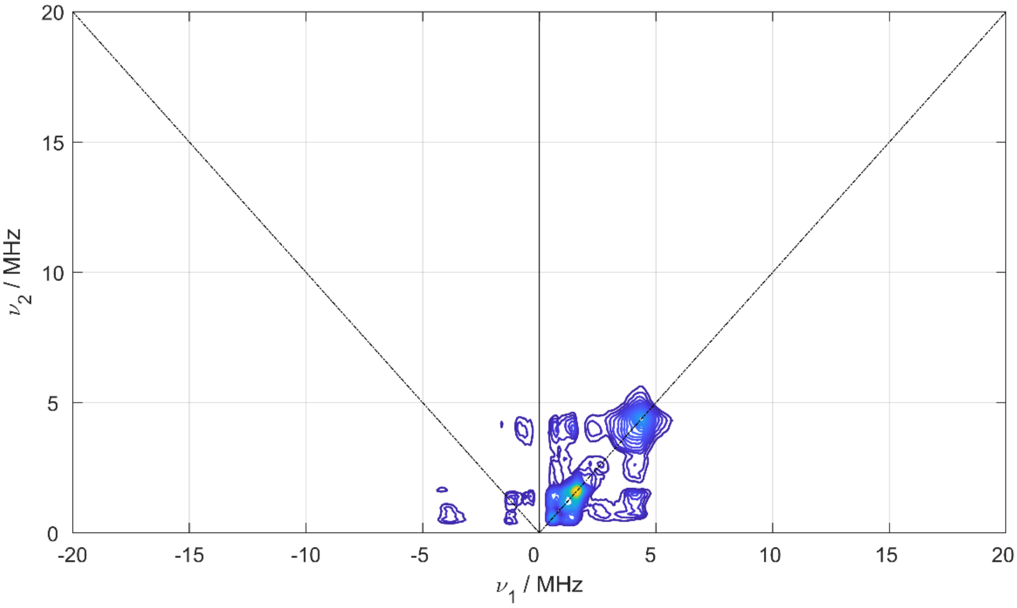

HYSCORE with 50 molar equivalents of Cu^{II}

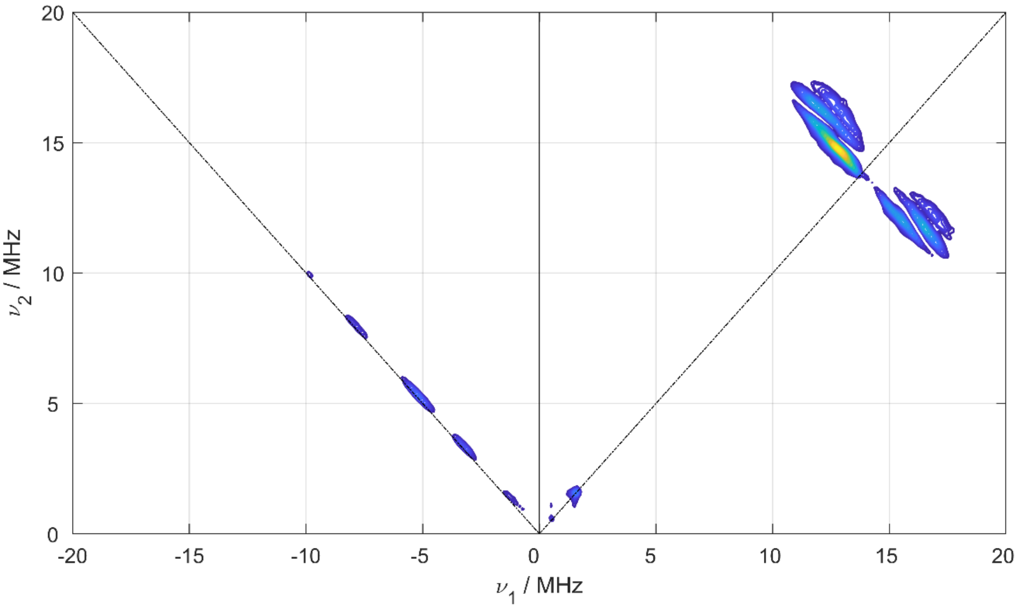

Figure S11, continued. Individual HYSCORE spectra. From top to bottom: 1 / 5 / 10 / 15 / 20 / 50 / 100 / 200 / 400 equivalents Cu^{II} and control (Cu^{II} in Tris buffer). See also Figure 4C in the manuscript.

HYSCORE with 100 molar equivalents of Cu^{II}

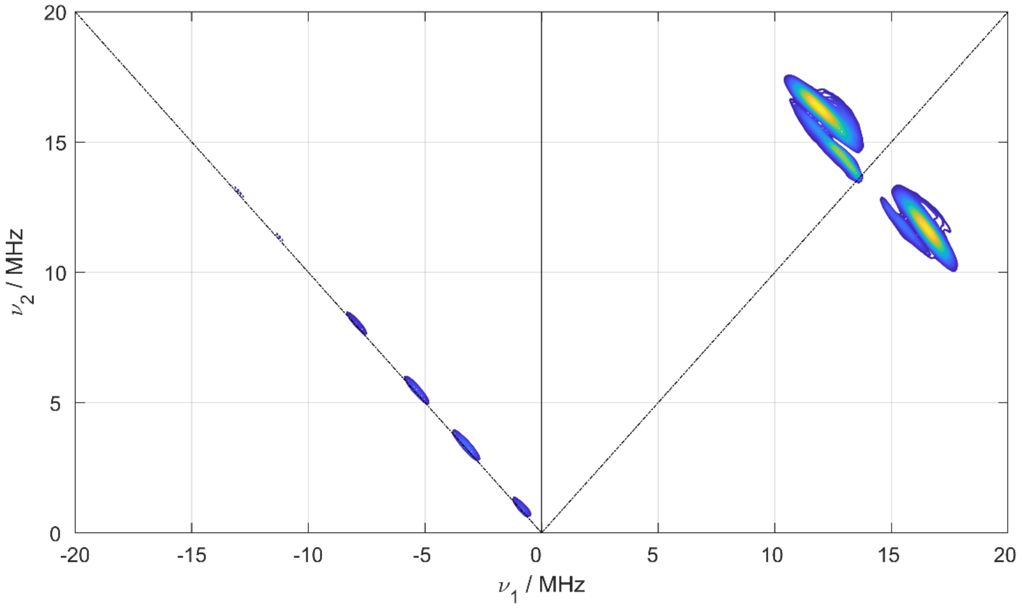

HYSCORE with 200 molar equivalents of Cu^{II}

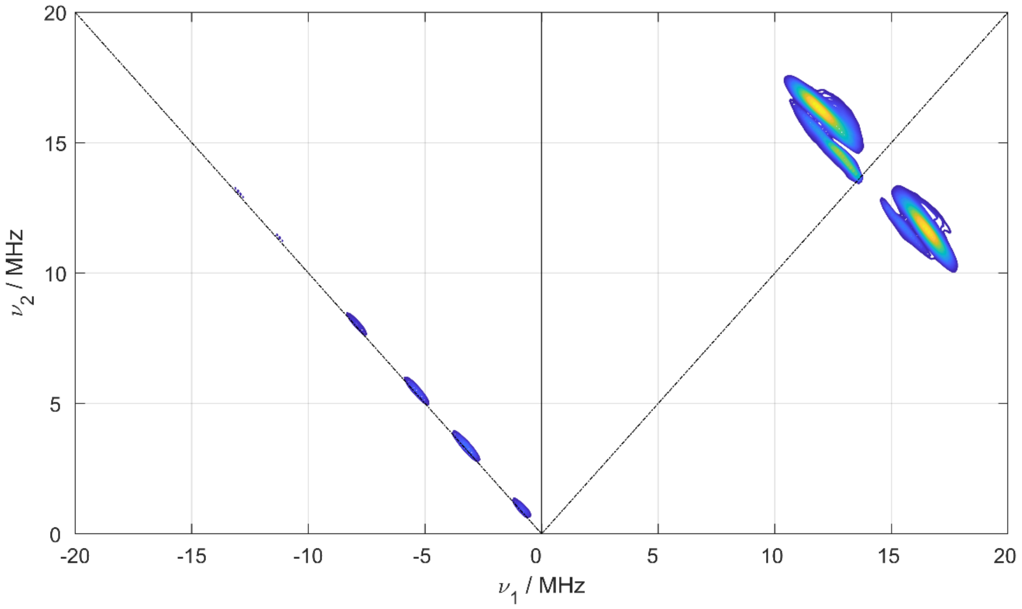

Figure S11, continued. Individual HYSCORE spectra. From top to bottom: 1 / 5 / 10 / 15 / 20 / 50 / 100 / 200 / 400 equivalents Cu^{II} and control (Cu^{II} in Tris buffer). See also Figure 4C in the manuscript.

HYSCORE with 400 molar equivalents of Cu^{II}

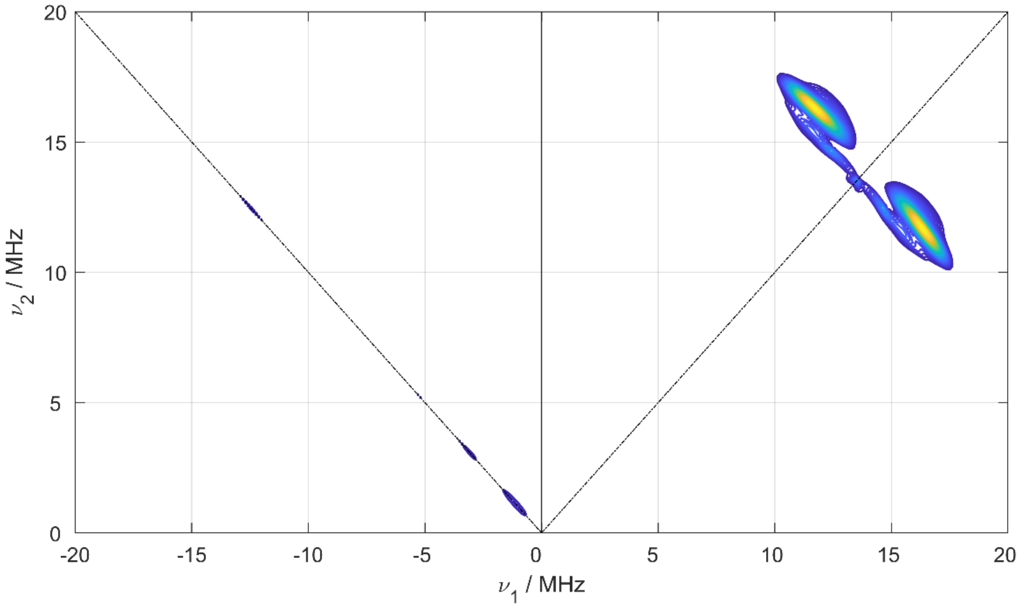

HYSCORE control sample with 1 molar equivalent of Cu^{II} (Cu^{II} in Tris buffer)

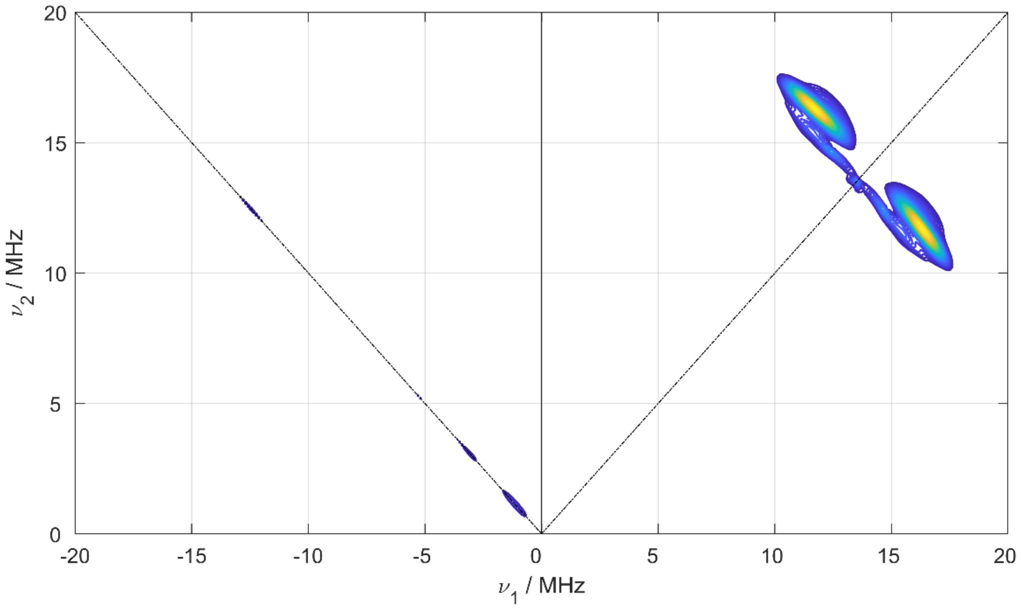

Figure S11, continued. Individual HYSCORE spectra. From top to bottom: 1 / 5 / 10 / 15 / 20 / 50 / 100 / 200 / 400 equivalents Cu^{II} and control (Cu^{II} in Tris buffer). See also Figure 4C in the manuscript.

In an attempt to visualise also the weak two-nitrogen double quantum transitions (visible in the ESEEM spectra at ~8 MHz)[22-23] we re-processed the 5 to 20 molar equivalents Cu^{II} HYSCORE spectra allowing more noise. This processing revealed weak but visible peaks at the expected positions, shown below in the projection contour plots, with strongest peaks observed at 15 molar eq. Cu^{II}, in line with our other observations.

HYSCORE with 5 molar equivalents of Cu^{II}

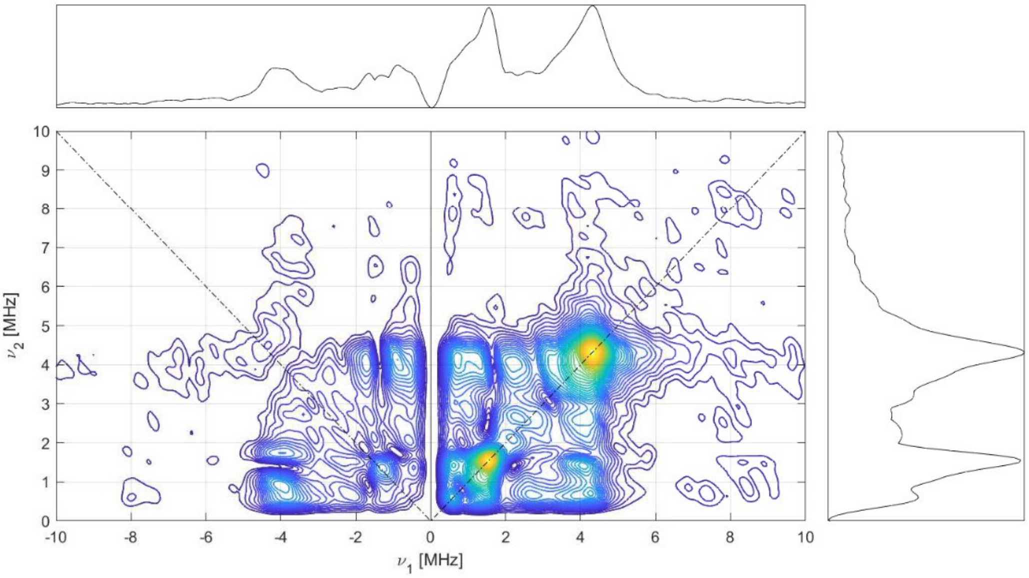

HYSCORE with 10 molar equivalents of Cu^{II}

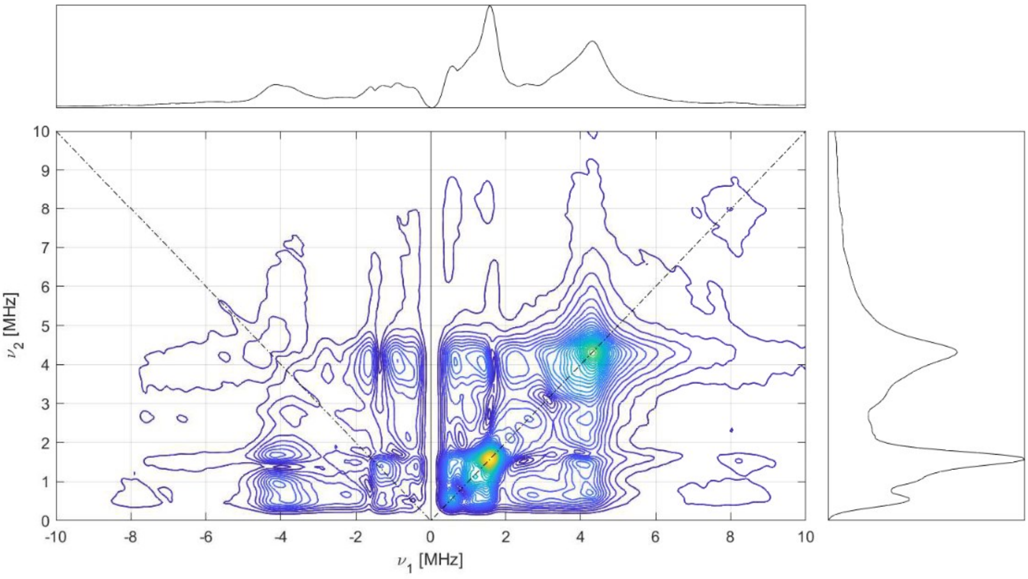

Figure S12. Individual HYSCORE spectra processed with different contour level settings to allow observation of the weak two-nitrogen double quantum transitions (~8 MHz). From top to bottom: 5, 10, 15, and 20 molar equivalents of Cu^{II}, with the peak most clearly seen for 15.

HYSCORE with 15 molar equivalents of Cu^{II}

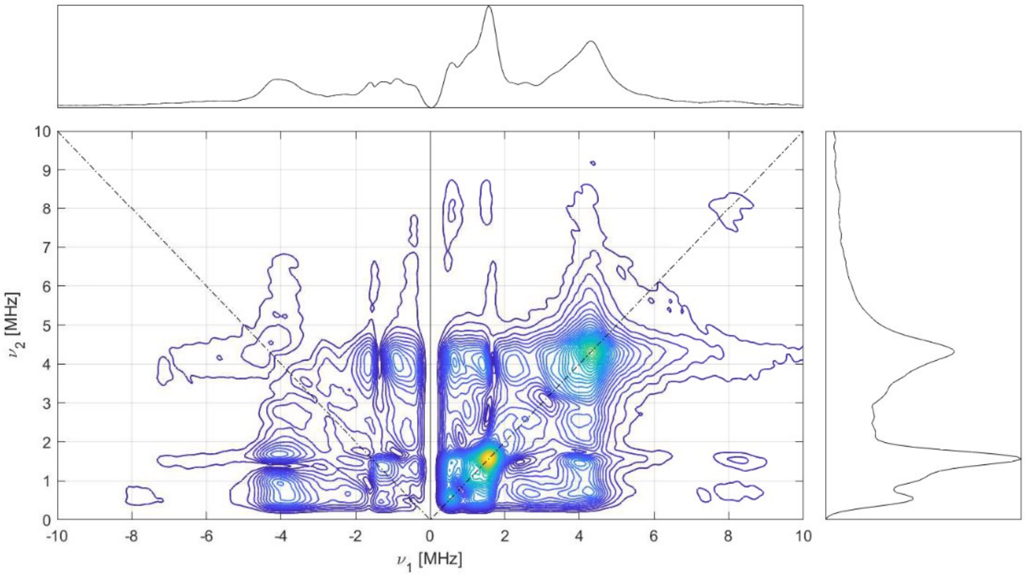

HYSCORE with 20 molar equivalents of Cu^{II}

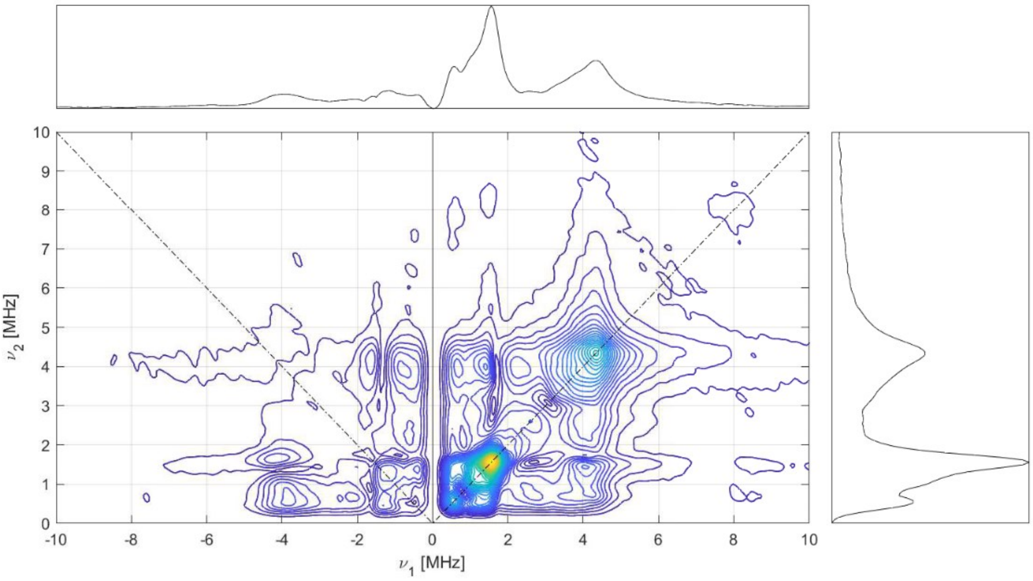

Figure S12, continued. Individual HYSCORE spectra processed with different contour level settings to allow observation of the weak two-nitrogen double quantum transitions (~8 MHz). From top to bottom: 5, 10, 15, and 20 molar equivalents of Cu^{II}, with the peak most clearly seen for 15.

5) Pulse dipolar EPR

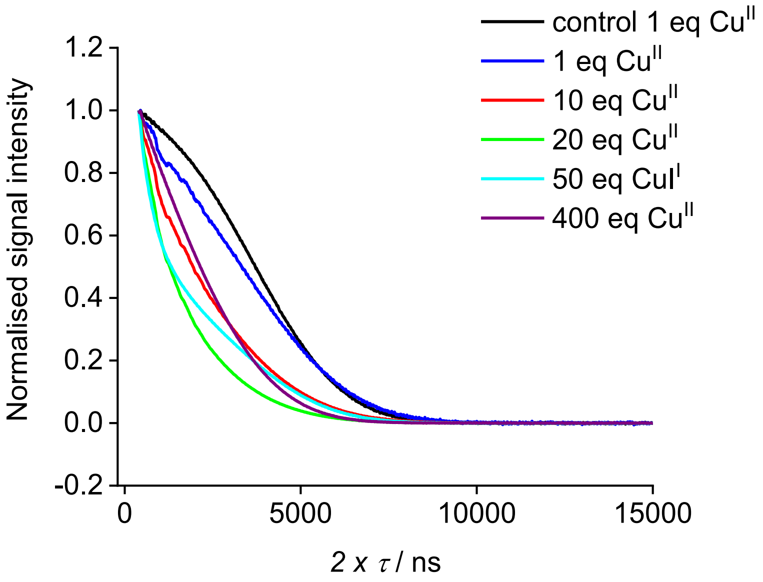

Figure S13. Two-pulse echo decay for selected samples. Note the marked step from 20 to 50 molar equivalents of Cu^{II}, with an additional component appearing in the decay.

Molar eq Cu^{II}	T_m	Stretching exponent	1/e time	1/(2e^2) time
	[μs]		[μs]	[μs]
1 (control)	2.1372	2.0000	2.200	1.472
1	2.0843	1.8605	2.072	1.488
10	1.2405	1.2203	1.320	1.128
20	0.7225	1.0000	0.888	0.828
50	1.0129	1.0000	1.064	1.096
400	1.3180	1.5565	1.360	1.040

Table S4. Overview of corresponding estimated T_m (~T_2) values under the stretched exponential approximation for Cu^{II}.

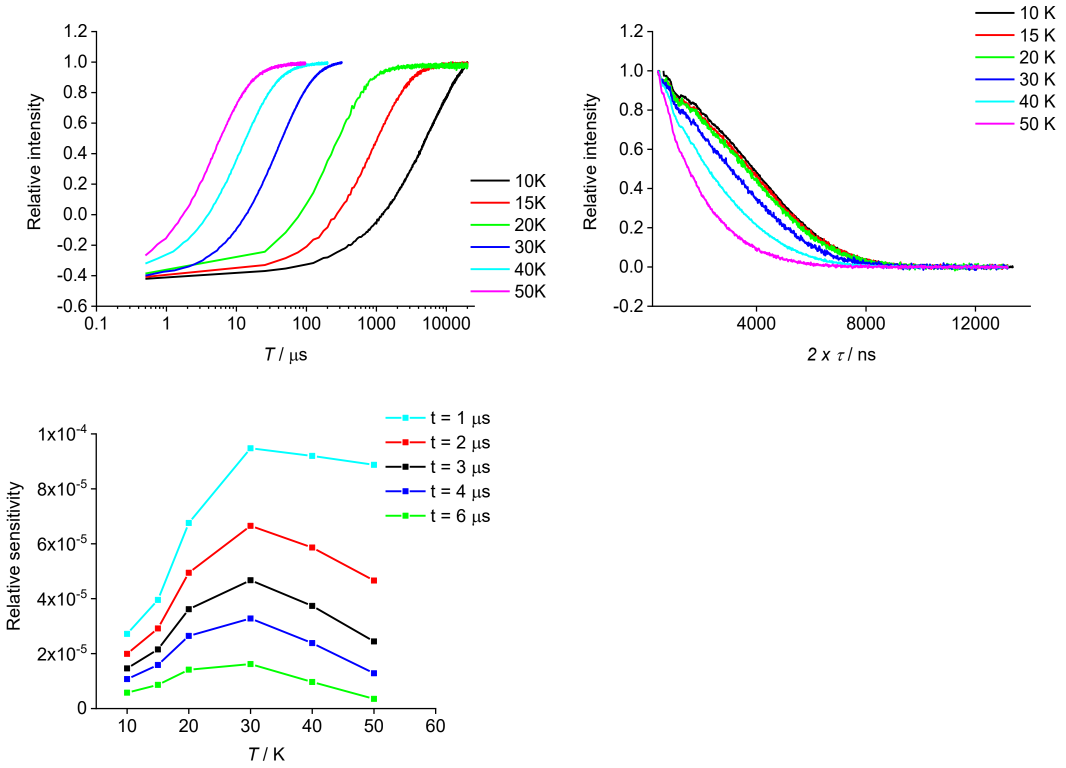

Figure S14. PELDOR temperature optimisation. All optimisation experiments were performed with HRG + 2 molar equivalents of Cu^{II} and 50% ethylene glycol for cryoprotection. T_1 is measured with a 3-pulse inversion recovery experiment (top left), T_m with a 2-pulse decay experiment (top right). The optimum temperature was derived from the relative sensitivity per temperature (bottom row) taking into account the Boltzmann factor, the temperature-dependent T_m (or T_2; i.e., how fast is the loss of coherence), and averaging (i.e., how fast do populations re-equilibrate) as described previously.[3] An optimum temperature of 30 K was determined for PELDOR measurements from these relaxation data.

**Temperature**	T_m	**Stretching exponent**	**1/e time**	**1/(2e^2) time**
[K]	[μs]		[μs]	[μs]
10	2.3482	2.0000	2.348	1.610
15	2.3193	2.0000	2.296	1.584
20	2.2659	2.0000	2.208	1.556
30	1.9623	1.7543	1.952	1.396
40	1.4264	1.3962	1.480	1.192
50	0.8656	1.1164	1.016	0.876

Table S5. Overview of corresponding estimated T_m (~T_2) values under the stretched exponential approximation for Cu^{II}.

**Temperature**	***T*₁ (mono-exp.)**	**1/e time**	**1/(2e²) time**
[K]	[μs]	[μs]	[μs]
10	5702 (5639, 5764)	4776	5463
15	1125 (1115, 1135)	951	1138
20	298 (295, 301)	276	326
30	45.0 (44.8, 45.3)	43.7	47.3
40	14.7 (14.6, 14.8)	14.0	15.3
50	6.32 (6.27, 6.37)	6.26	6.91

Table S6. Overview of corresponding estimated T_1 values under the mono-exponential approximation for Cu^{II}, the 95% confidence bounds are given in brackets.

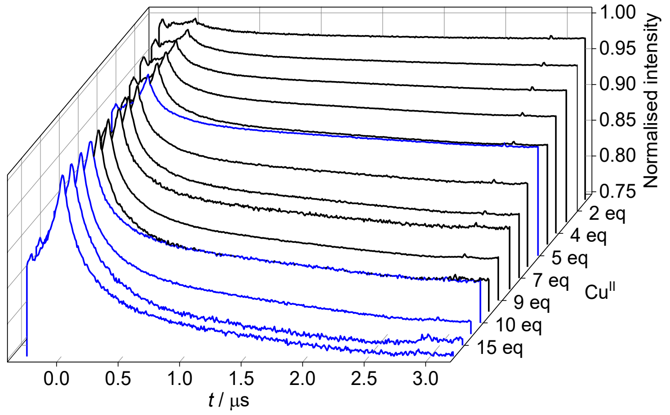

Figure S15. PELDOR raw traces of the pseudo-titration shown in a waterfall plot. A second batch of purified protein, shown in blue, was prepared to generate the higher equivalent samples (12, 15, and 20 molar equivalents of Cu^{II}); additionally, two samples were prepared for comparison with the first batch of protein (5 and 10 molar equivalents of Cu^{II}). Corresponding background-corrected data and modulation depths are shown in manuscript Figure 5.

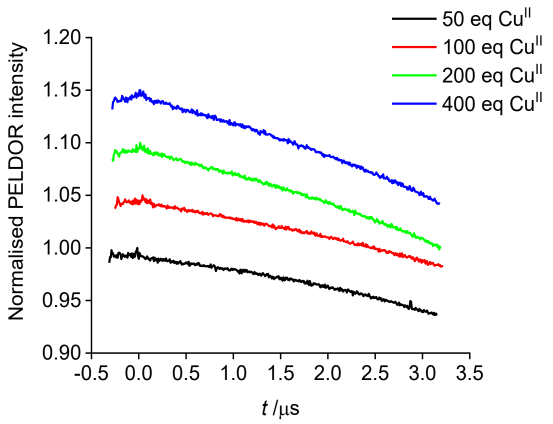

Figure S16. PELDOR raw traces of the high equivalent samples (50, 100, 200, and 400 equivalents of Cu^{II}). Traces are y-stacked for convenience. No modulation can be observed in any of the traces. Note that without the 'drowning out' effect the 50 eq. Cu^{II} sample should show Δ = 0.08 (0.4 x 0.2 + 0.6 x 0), which is not observed. No distributions could be obtained from the high equivalent samples not displaying any modulation.

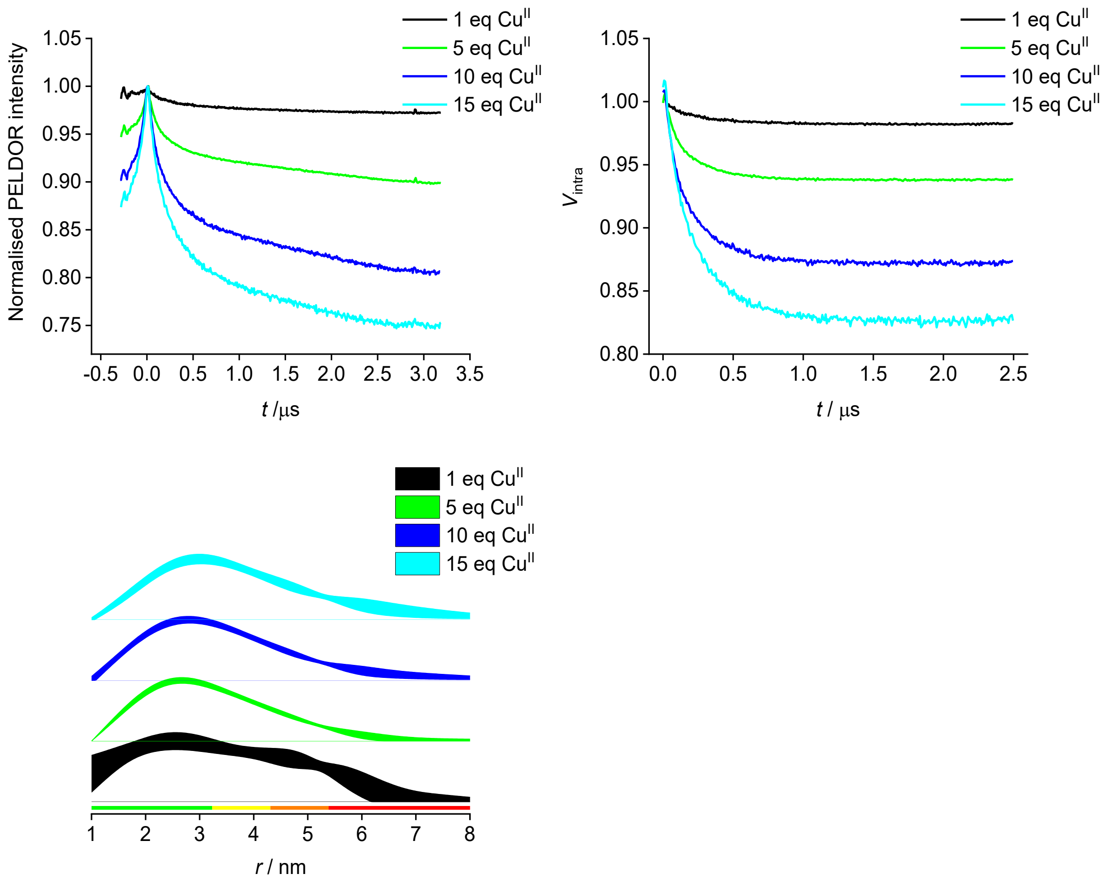

Figure S17. Exemplary PELDOR data for HRG with 1, 5, 10, and 15 molar equivalents of Cu^{II} added. Raw and background-corrected data are shown in the left and middle graphs, respectively. Corresponding y-stacked distance distributions are given to the right. Shown are the 95% confidence estimates (± 2σ) of the distance distributions as obtained by statistical analysis. Colour bars represent reliability ranges as described in the DeerAnalysis[8] manual (green: shape reliable; yellow: mean and width reliable; orange: mean reliable: red: no quantification possible).

As shown above, PELDOR experiments yielded very broad distance distributions that we refrained from quantifying and that did not change significantly within confidence intervals between 1 and 20 equivalents of Cu^{II} added.

6) Speciation model to simulate PELDOR modulation depths in HRG

Constructing a multi-site binding polynomial

We are assuming a speciation model with two different metal ion binding sites for the HRG, each with different affinities reflecting high and lower affinity sites. Simulation of empirical modulation depths observed in the HRG + Cu^{II} PELDOR pseudo-titration series requires a mathematical description of the protein-ligand binding equilibria of each species in solution, which can be achieved via a general multi-site binding polynomial.[24]

The fractional population of each species, the macroscopic speciation vector, f_i, is a function of the following parameters: total protein concentration, $[P]_0$, total ligand concentration, $[M]_0$, dissociation constants $K_{D1}$and K_{D2}, and the number of high affinity, n, and low affinity, m, sites. These fractional macroscopically-bound populations are significant in the simulation of PELDOR modulation depths because each species will contribute to the observed modulation depth with a weighting proportional to the product of their relative population, and the number of spins present in that species:

$$\Delta_{PELDOR} = 1 - \left(\frac{\sum_{i=1}^{N} f_i \times (1 - \lambda_{PELDOR})^{N-1} \times N}{\sum_{i=1}^{N} f_i \times N} \right)$$

Where f is as defined above, λ_{PELDOR} is the inversion efficiency of the pumping pulse and N is the total number of spins in the system. The unmodulated echo contributions $(1 - \lambda_{PELDOR})^{N-1}$ are averaged for all species with i = 1 to N Cu^{II} ions bound taking into account the increase in Cu^{II} signal and normalising by their contribution to the signal at zero time.[9] PELDOR modulation depths were simulated using MATLAB, and mean square error was used as a metric for simulation quality:

$$mse = mean\left(\left(\Delta_{sim} - \Delta_{exp} \right)^2 \right)$$

Exploratory simulations of PELDOR modulation depths

Cu^{II}-Cu^{II} Q-band PELDOR measurements were performed on rHRG at 125 µM protein concentration, and with varying equivalents of Cu^{II}, ranging in concentration from 125-2500 µM. Previous literature, and empirical ITC data suggested there are 12 high-affinity ligand-binding sites (*n* = 12), with a K_D (at 235 K) of 5×10^{-8}. The extrapolation of K_D to low temperature was facilitated by application of the van't Hoff equation and is made necessary because the binding kinetics are fast with respect to the flash-freezing, and so EPR measurements reflect the binding equilibrium at the freezing-point temperature. The inversion efficiency (λ) of a 16 ns rectangular pump pulse respective of Cu^{II} spectra at Q-band frequency, and with an offset of ~150 MHz was demonstrated to be 1-2%.[25] For the subsequent

simulations, inversion efficiency was approximated as 1.5% (λ = 0.015), unless otherwise stated. It should be noted that for the K_D and number of low affinity sites, there was little literature or empirical data to corroborate accurate values.

Since approximate values are available for all other parameters, we first investigated the influence of the number, and K_D of the low affinity sites. The experimental modulation depths and 95% confidence intervals are given in Table S7, with the corresponding Cu^{II} concentrations. Figure S18 shows error surfaces for value pairs of (m, K_{D2}) when comparing simulated values to the empirical data, and clearly demonstrates the relative insensitivity of the simulation to the number and affinity of the second class of sites.

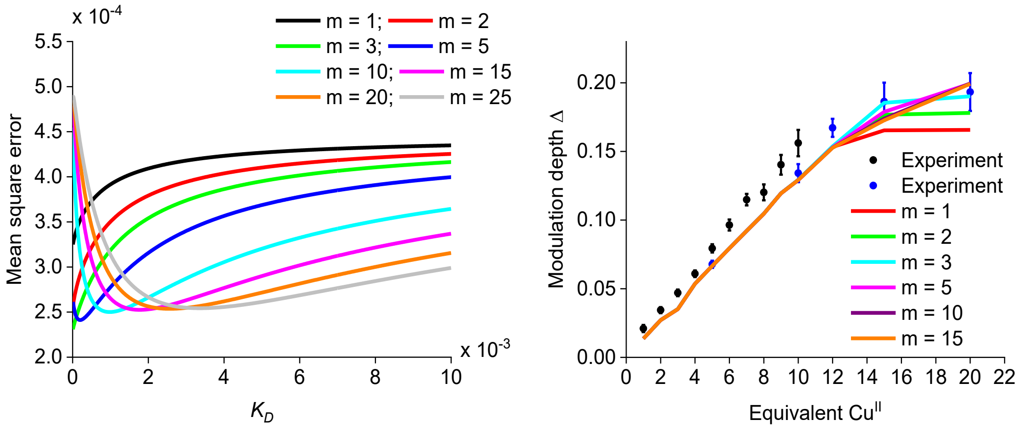

Figure S18. A comparative plot of the mean square error as a function of K_D for fixed values of m (left), and mean experimental (with 2 × σ error; black: first batch, blue: second batch of HRG) and simulated modulation depths corresponding to the local minimum for each (m, K_D) pair (right). Simulations are performed with the following parameters: $[P]_0$ = 1.25 x 10^{-4} M, $[M]_0$ = [0.125, 0.250, 0.375, 0.500, 0.675, 0.750, 0.875, 1.000, 1.125, 1.250, 1.500, 1.875, 2.500] x 10^{-3} M, n = 12, K_{D1} = 5 x 10^{-8}, m = [1, 2, 3, 5, 10, 15], K_{D2} = [10, 20, 180, 690, 2000, 3500] x 10^{-6} and λ = 0.015. NB: For m = 20 and 25, simulations are not shown.

Table S8 gives the error minima for value pairs of (m, K_{D2}), and indicates that as the number of low-affinity sites increases, the affinity correspondingly decreases, as would be expected for a constant modulation depth. While the global error minimum is at (m = 3, K_{D2} = 1.0 × 10^{-5}), it is seen that the error minimum is not particularly pronounced, and experimental data is also reasonably described by m = 5, 10 and 15.

Cu^{II} concentration (μM)	Experimental PELDOR modulation depth (Δ_{PELDOR})	Mean Δ_{PELDOR} (after validation)	Δ_{PELDOR} error ($2 \times \sigma$)
125	0.021	0.021	0.0026
250	0.035	0.034	0.0022
375	0.047	0.047	0.0024
500	0.061	0.061	0.0024
625	0.080 / 0.068	0.079 / 0.068	0.0030 / 0.0026
750	0.097	0.096	0.0040
875	0.115	0.115	0.0042
1000	0.120	0.120	0.0058
1125	0.141	0.140	0.0072
1250	0.157 / 0.135	0.156 / 0.134	0.0096 / 0.0066
1500	0.168	0.167	0.0066
1875	0.187	0.186	0.0138
2500	0.193	0.193	0.0138

Table S7. The experimental Cu^{II}-Cu^{II} PELDOR modulation depths from the corresponding HRG pseudo-titration.

Error Surface Minimum / arb. unit	*m*	K_D / × 10^{-6}
3.3 x 10^{-4}	1	10
2.6 x 10^{-4}	2	10
2.3 x 10^{-4}	3	10
2.4 x 10^{-4}	5	200
2.5 x 10^{-4}	10	980
2.5 x 10^{-4}	15	1800
2.5 x 10^{-4}	20	2500
2.5 x 10^{-4}	25	3300

Table S8. The minimum mean square error of each trace shown in Figure S18 (left) and the corresponding optimum value of K_D.

For the first 12 equivalents of Cu^{II} added to the HRG, the modulation depth is approximately linearly increasing and implies that binding is quantitative (i.e., $n \geq 12$ and $K_{D1} << 1.25 \times 10^{-4}$). Since Δ continues to increase for 15 and 20 molar equivalents of Cu^{II}, this implies that HRG continues to bind Cu^{II}, and that low affinity sites are not fully saturated in the regime of Cu^{II} concentrations initially investigated.

Therefore, this suggests that $m \geq 8$ ($m = 8$ presupposes that binding is quantitative, which is unlikely given that the optimal low-affinity K_D values predicted by the simulation (for $m \geq 5$) are > 1.25×10^{-4}). It is more probable that the true value of m is significantly higher than 8, since this would account for the continuously increasing Δ and the non-linearity of the increase for 15 and 20 equivalents of Cu^{II} (many sites being sparsely populated), however one cannot use the simulations to make precise inferences about the number and K_D of the low affinity sites.

Contrarily, it can be shown that to satisfactorily describe the observed data, particularly for the first 12 equivalents, $n \geq 12$, as illustrated in Figure 5 in the main text.

This is highly consistent with the previous literature and empirical ITC data, and values greater than $n = 12$ lead to a simulated modulation depth profile which begins to plateau at higher concentrations of Cu^{II} and therefore overestimate Δ at 20 equivalents. It should also be noted that regardless of the K_{D1} chosen for the simulation, the error function is always minimized for $n = 12$. In light of CW EPR measurements that indicated sustained Cu^{II}-binding up to 100 equivalents of Cu^{II} with respect to HRG protein, further simulation was performed using 100 low-affinity binding sites ($m = 100$), with a dissociation constant of ~5.0×10^{-2}. Results are shown below.

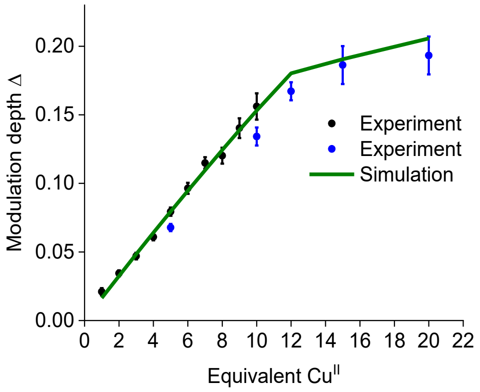

Figure S19: A plot of experimental (with $2 \times \sigma$ error; black: first batch, blue: second batch of HRG) vs simulated modulation depths. Simulations were performed with the following other parameters: $[P]_0 = 1.25 \times 10^{-4}$ M, $[M]_0$ = [0.125, 0.250, 0.375, 0.500, 0.675, 0.750, 0.875, 1.000, 1.125, 1.250, 1.500, 1.875, 2.500] x 10^{-3} M, $n = 12$, $m = 100$, $K_{D1} = 5 \times 10^{-8}$, $K_{D2} = 5.0 \times 10^{-2}$ and $\lambda = 0.018$.

7) References

(1) Oranges, M.; Wort, J. L.; Fukushima, M.; Fusco, E.; Ackermann, K.; Bode, B. E. Pulse Dipolar Electron Paramagnetic Resonance Spectroscopy Reveals Buffer-Modulated Cooperativity of Metal-Templated Protein Dimerization. *J. Phys. Chem. Lett.* **2022,** *13*, 7847-7852. DOI: 10.1021/acs.jpclett.2c01719

(2) Ferreira, C. M. H.; Pinto, I. S. S.; Soares, E. V.; Soares, H. M. V. M. (Un)Suitability of the Use of pH Buffers in Biological, Biochemical and Environmental Studies and their Interaction with Metal Ions - a Review. *Rsc Adv.* **2015,** *5*, 30989-31003. DOI: 10.1039/c4ra15453c

(3) Jeschke, G.; Polyhach, Y. Distance Measurements on Spin-Labelled Biomacromolecules by Pulsed Electron Paramagnetic Resonance. *Phys. Chem. Chem. Phys.* **2007,** *9*, 1895-1910. DOI: 10.1039/b614920k

(4) Larsen, R. G.; Singel, D. J. Double Electron-Electron Resonance Spin-Echo Modulation - Spectroscopic Measurement of Electron-Spin Pair Separations in Orientationally Disordered Solids. *J. Chem. Phys.* **1993,** *98*, 5134-5146. DOI: Doi 10.1063/1.464916

(5) Milov, A. D.; Salikhov, K. M.; Shirov, M. D. Application of ELDOR in Electron-Spin Echo for Paramagnetic Center Space Distribution in Solids. *Fiz. Tverd. Tela* **1981,** *23*, 975-982.

(6) Pannier, M.; Veit, S.; Godt, A.; Jeschke, G.; Spiess, H. W. Dead-Time Free Measurement of Dipole-Dipole Interactions between Spins. *J. Magn. Reson.* **2000,** *142*, 331-340. DOI: 10.1006/jmre.1999.1944

(7) Kerry, P. S.; Turkington, H. L.; Ackermann, K.; Jameison, S. A.; Bode, B. E. Analysis of Influenza A Virus NS1 Dimer Interfaces in Solution by Pulse EPR Distance Measurements. *J. Phys. Chem. B* **2014,** *118*, 10882-8. DOI: 10.1021/jp508386r

(8) Jeschke, G.; Chechik, V.; Ionita, P.; Godt, A.; Zimmermann, H.; Banham, J.; Timmel, C. R.; Hilger, D.; Jung, H. DeerAnalysis2006 - a Comprehensive Software Package for Analyzing Pulsed ELDOR Data. *Appl. Magn. Reson.* **2006,** *30*, 473-498. DOI: 10.1007/BF03166213

(9) Ackermann, K.; Pliotas, C.; Valera, S.; Naismith, J. H.; Bode, B. E. Sparse Labeling PELDOR Spectroscopy on Multimeric Mechanosensitive Membrane Channels. *Biophys. J.* **2017,** *113*, 1968-1978. DOI: 10.1016/j.bpj.2017.09.005

(10) Mims, W. B. Envelope Modulation in Spin-Echo Experiments. *Phys. Rev. B* **1972,** *5*, 2409-2419. DOI: 10.1103/PhysRevB.5.2409

(11) Mims, W. B. Amplitudes of Superhyperfine Frequencies Displayed in the Electron-Spin-Echo Envelope. *Phys. Rev. B* **1972,** *6*, 3543-3545. DOI: 10.1103/PhysRevB.6.3543

(12) Rowan, L. G.; Hahn, E. L.; Mims, W. B. Electron-Spin-Echo Envelope Modulation. *Phys. Rev.* **1965,** *137*, A61-A71. DOI: 10.1103/PhysRev.137.A61

(13) Volkov, A.; Dockter, C.; Bund, T.; Paulsen, H.; Jeschke, G. Pulsed EPR Determination of Water Accessibility to Spin-Labeled Amino Acid Residues in LHCIIb. *Biophys. J.* **2009,** *96*, 1124-1141. DOI: 10.1016/j.bpj.2008.09.047

(14) Van Doorslaer, S. Hyperfine Spectroscopy: ESEEM. *eMagRes* **2017,** *6*, 51-69. DOI: 10.1002/9780470034590.EMRSTM1517

(15) Fabregas Ibanez, L.; Soetbeer, J.; Klose, D.; Tinzl, M.; Hilvert, D.; Jeschke, G. Non-Uniform HYSCORE: Measurement, Processing and Analysis with Hyscorean. *J. Magn. Reson.* **2019,** *307*, 106576. DOI: 10.1016/j.jmr.2019.106576

(16) Goldfarb, D. ELDOR-Detected NMR. *eMagRes* **2017,** *6*, 101-114. DOI: 10.1002/9780470034590.emrstm1516

(17) Schosseler, P.; Wacker, T.; Schweiger, A. Pulsed ELDOR Detected NMR. *Chem. Phys. Lett.* **1994,** *224*, 319-324. DOI: 10.1016/0009-2614(94)00548-6

(18) Stoll, S.; Schweiger, A. EasySpin, a Comprehensive Software Package for Spectral Simulation and Analysis in EPR. *J. Magn. Reson.* **2006,** *178*, 42-55. DOI: 10.1016/j.jmr.2005.08.013

(19) Cox, N.; Nalepa, A.; Lubitz, W.; Savitsky, A. ELDOR-Detected NMR: A General and Robust Method for Electron-Nuclear Hyperfine Spectroscopy? *J. Magn. Reson.* **2017,** *280*, 63-78. DOI: 10.1016/j.jmr.2017.04.006
(20) Muhoberac, B. B.; Burch, M. K.; Morgan, W. T. Paramagnetic Probes of the Domain Structure of Histidine-Rich Glycoprotein. *Biochemistry* **1988,** *27*, 746-52. DOI: 10.1021/bi00402a038
(21) Silva, K. I.; Michael, B. C.; Geib, S. J.; Saxena, S. ESEEM Analysis of Multi-Histidine Cu(II)-Coordination in Model Complexes, Peptides, and Amyloid-Beta. *J. Phys. Chem. B* **2014,** *118*, 8935-44. DOI: 10.1021/jp500767n
(22) Cunningham, T. F.; Putterman, M. R.; Desai, A.; Horne, W. S.; Saxena, S. The Double-Histidine Cu^{2+}-Binding Motif: a Highly Rigid, Site-Specific Spin Probe for Electron Spin Resonance Distance Measurements. *Angew. Chem. Int. Ed.* **2015,** *54*, 6330-4. DOI: 10.1002/anie.201501968
(23) Klose, D.; Vemulapalli, S. P. B.; Richman, M.; Rudnick, S.; Aisha, V.; Abayev, M.; Chemerovski, M.; Shviro, M.; Zitoun, D.; Majer, K.; Wili, N.; Goobes, G.; Griesinger, C.; Jeschke, G.; Rahimipour, S. Cu^{2+}-Induced Self-Assembly and Amyloid Formation of a Cyclic D,L-Alpha-Peptide: Structure and Function. *Phys. Chem. Chem. Phys.* **2022,** *24*, 6699-6715. DOI: 10.1039/d1cp05415e
(24) Wort, J. L.; Ackermann, K.; Norman, D. G.; Bode, B. E. A General Model to Optimise Cu^{II} Labelling Efficiency of Double-Histidine Motifs for Pulse Dipolar EPR Applications. *Phys. Chem. Chem. Phys.* **2021,** *23*, 3810-3819. DOI: 10.1039/D0CP06196D
(25) Wort, J. L.; Ackermann, K.; Giannoulis, A.; Stewart, A. J.; Norman, D. G.; Bode, B. E. Sub-Micromolar Pulse Dipolar EPR Spectroscopy Reveals Increasing Cu(II) -labelling of Double-Histidine Motifs with Lower Temperature. *Angew. Chem. Int. Ed.* **2019,** *58*, 11681-11685. DOI: 10.1002/anie.201904848
